# Association between the *ABCA1* (R219K) polymorphism and lipid profiles: a meta-analysis

**DOI:** 10.1038/s41598-021-00961-9

**Published:** 2021-11-05

**Authors:** Zhangyan Shi, Yajie Tian, Ze Zhao, Yufei Wu, Xiuxia Hu, Junlin Li, Qianliang Chen, Yan Wang, Caiyan An, Kejin Zhang

**Affiliations:** 1 College of Life Science, Northwest University, Xi’an, 710069 China; 2 College of Medicine, Northwest University, Xi’an, 710069 China; 3 Shaanxi Key Laboratory of Biomedicine, College of Life Science, Northwest University, Xi’an, 710069 China; 4 Clinical Medical Research Center of the Affiliated Hospital, Inner Mongolia Medical College, Hohhot, 010050 China; 5 Institute of Population and Health, College of Life Science, Northwest University, Xi’an, 710069 China

**Keywords:** Genetics, Biomarkers, Diseases

## Abstract

Conflicting evidence was found about the relationship between lipid profiles and R219K polymorphism in adenosine triphosphate-binding cassette exporter A1 (*ABCA1*) gene. In this study, four meta-analyses were conducted to assess the effect of R219K on lipid levels, including high-density lipoprotein cholesterol (HDLC), low-density lipoprotein cholesterol, total cholesterol, and triglycerides (TG). A total of 125 samples of 87 studies (about 60,262 subjects) were included. The effect of each study was expressed using the standard mean difference (SMD) and 95% confidence interval (95% CI) and pooled by meta-analysis in the random-effects model. Subgroup and meta-regression analyses were conducted to explore potential heterogeneity sources. The overall pooled effect showed the following results. (1) The R219K was significantly associated with HDLC level (SMD = − 0.25 mmol/L, 95%CI − 0.32 to − 0.18, *z* = − 6.96, *P* < 0.01, recessive genetic model). People with different genotypes had significantly different HDLC levels under the recessive, codominant and dominant genetic models (all *P*s < 0.01). (2) A weak and indeterminate relationship between R219K and TG level was observed (SMD = 0.18 mmol/L, 95%CI 0.06–0.30, *z* = 3.01, *P* < 0.01, recessive genetic model). These findings suggested that R219K was associated with HDLC and TG levels, which might implicate a promising clinical application for lipid-related disorders, though the influences of race, health status, BMI, and other heterogeneity sources should be considered when interpreting current findings. The protocol was registered at PROSPERO (registration number: CRD42021231178).

## Introduction

An optimal blood lipid level is important. Abnormalities in blood lipid levels are causally linked to several common human diseases, such as cardiovascular disease (CVD), diabetes, obesity and stroke^1–6^. The lipid profile generally serves as an initial screening tool for lipid abnormalities and an important predictor of the above diseases. Investigations to explore the sources that contribute to the difference of lipid levels amongst individuals have attracted the interest of clinicians and researchers.

The lipid levels in the blood, which are usually presented by four traditional lipids (i.e. high-density lipoprotein cholesterol, HDLC; low-density lipoprotein cholesterol, LDLC; total cholesterol, TC; and triglycerides, TG), have high heritability. Approximately 10–15% of the variances amongst individual blood lipid levels can be explained by genetic effects^7^. Consequently, understanding the genetic architecture and regulation of lipid profiles will help in predicting, monitoring and treating the above human diseases^8^. To date, many genes which involved in lipid metabolism have been identified to be associated with variations in individual lipid levels^9,10^.

The ABCA1 is an ATP-binding cassette (ABC) subfamily A transporter. Genetic variants of the *ABCA1* gene are generally believed to cause individual differences in lipid levels^11–13^ because of its important role in controlling the circulating lipoprotein levels between cellular and extracellular media^14^. An arg219-to-lys (R219K, rs2230806) polymorphism in the seventh exon of the *ABCA1* gene has been extensively studied, although its biological function remains not fully understood. The association between R219K and lipid levels is often used as an endophenotype of patients and is combined with clinical diagnosis to help identify the causal genetic factors of human diseases, including hypercholesterolaemia^15^, heart disease^16^ and CVD^13,17^. However, those reported correlations remain inconsistent and are ambiguous.

The links observed between the R219K polymorphism and risk of above diseases have resulted in other questions from clinicians and scientists. (1) Do the genotypes of R219K significantly contribute to the difference in human blood lipid levels no matter in patients or general/healthy population? This issue means that individuals born with a favourable genotype have already some advantages in CVD, diabetes, obesity, stroke and other diseases events compared with those born with other genotypes^18^; (2) the difference in the effect of R219K on lipids level between the general population and patients is due to abnormal levels of certain lipids. Given that the significant difference in the effect between two populations, genetic detection is valuable and promising for the above diseases’ diagnosis, monitoring and strategic therapeutic approach design; (3) the influence of potential modulators on the correlation between R219K and lipid profile should be estimated. The effect of R219K on the level of lipids is still not well defined because every study is evaluated in various cohorts (e.g. race, age, sex, body mass index (BMI), healthy or with all kind of diseases and others). Previous meta-analyses^19–34^ are mainly emphasised the genetic variants of R219K and the risk of diseases rather than the consistency of their relationship amongst the general population. Furthermore, results from new studies remain inconsistent with those based on previous meta-analyses. The difference of the correlations between R219K and lipid profile in patients and general population is not extensively discussed and estimated quantitatively.

Four updated meta-analyses of the correlations between R219K polymorphism and HDLC, LDLC, TC and TG levels are conducted to reach conclusive answers. This work aims to: (1) investigate the relationship between R219K polymorphism and individual lipid levels within both the general population and patients with common diseases; (2) assess the consistency of this relationship amongst different populations; and (3) estimate the influence of other external factors on this correlation.

## Methods

### Search strategy and criteria

**Figure 1:**
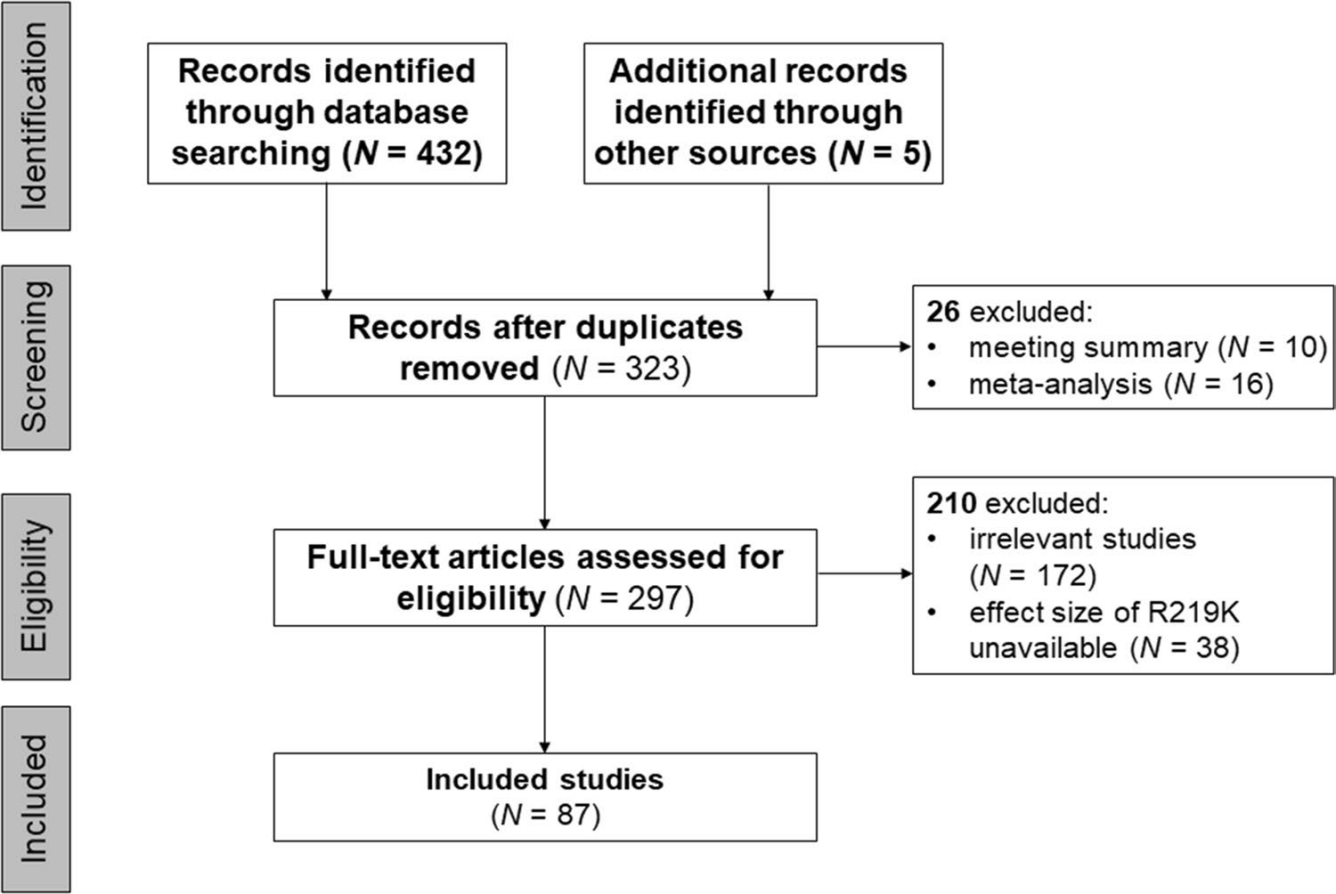
A literature reviewing for the relationship between R219K and lipid profiles.

This systematic review protocol was submitted for registration to the International Prospective Register of Systematic Reviews (http://www.crd.york.ac.uk/prospero) on 5 February 2021 and published on 8 March 2021 (Registration ID: CRD42021231178). Two authors (Shi Z and Tian Y) searched for studies published before 31 March 2021 and extracted useful data from the databases of BIOSIS, CENTRAL and Clinical trials, Derwent Innovations Index, EMBASE, InspecR, ISI Web of Science, KCI-Korean Journal Database, MEDLINE, PubMed, SciELO Citation Index and Scopus for papers in English and the Chinese National Knowledge Infrastructure and Wanfang databases for papers in Chinese. The full search strategy and literature terms used for searching the above databases are described in Fig. 1.

The inclusion criteria were as follows: (1) the mean lipids and standard deviations (SD) and/or standard errors (SE) were available for each group according to R219K genotypes; (2) at least one of the four variables (i.e. HDLC, LDLC, TC and TG) was available; (3) the frequency of R219K genotype and the deviation of genotypes from the Hardy–Weinberg equilibrium (HWE) was also described; and (4) other related information that can be used to calculate the effect size of R219K on the four lipid variables or the required data could be collected from other publications. Similarly, the exclusion criteria were as follows: (1) duplication and meeting summary; (2) no relevance of the R219K polymorphism on the *ABCA*1 gene; and (3) the original document was unavailable.

### Data extraction and quality assessment

Information extracted from the eligible literature included the following: (1) name of the first-author, time of publication and number of samples; (2) health status, ethnicity, BMI and mean age of subjects and sex (proportion of females in each sample); (3) mean lipid levels, standard deviation and/or standard error for different groups according to R219K genotype; and (4) other format data that could be used to calculate the effect size value. Required data were extracted and formatted as described in the online supplementary material (Supplementary Tables S1 and S2). The quality of all articles was evaluated by two authors (Shi and Tian) in accordance with the Newcastle–Ottawa Scale (NOS) assessment scale^35^. Comparing the frequencies of studies categorized by the median NOS score of included studies, two authors’ consensus assessments had high inter-rater reliability (*κ* = 0.81, *P* < 0.01). The deviation from the HWE for each sample’s distribution of R219K genotypes was assessed using the *HardyWeinberg* package^36^ and used as one of important index of NOS.

### Statistical analysis

We conducted meta-analysis by using two R programs of *meta* and *metaphor*^37,38^. The effect size of R219K on lipid levels in each study was presented in the standardised mean difference (SMD) and 95% confidence interval (95% CI) format since various measurements and methods were involved with different scales^39^. The random-effects model, which considered within- and between-study variances^40^, was adopted to estimate the pooled effect size of all studies because of the potential sources of heterogenous (e.g. different measurements of lipid levels, race, health status of subjects, age and NOS score) amongst eligible studies. Heterogeneity was assessed using the Cochran’s *Q*-statistic and *I*^2^ statistics^41^. Regarding R219K polymorphism’s inconsistent genetic structure in different population and diverse genetic models reports, three codominant models (RR vs. KK, RR vs. RK and RK vs. KK), dominant (RR + RK vs. KK), recessive (RR vs. RK + KK) and codominant (RK vs. RR + KK) models were used to estimate this variant’s effect size on human lipid levels.

The sources of heterogeneity were explored and their influences were assessed by (1) pooling the effect of each subgroup defined by categorical variables (e.g. race and health status) and (2) comparing the effects of the subgroups as recommended by Borenstein and Higgins^42^ and (3) estimating the influence of interested potential moderators on the effect of R219K. A meta-regression analysis was performed using the mixed-effects model (random-effects model within subgroups, fixed-effects model amongst subgroups)^43^. The differences of the effect of R219K on lipid profiles between groups were tested using the method of DerSimonian-Laird estimator for *tau*^2^^44^. Simultaneously, the omnibus test (*Q*_*M*_) and goodness of fit test (*Q*_*G*_) were carried out to access the influence of all target moderators on the effect and calculate whether other unknown moderators also contributed to the variance of effect of R219K on lipid profiles.

Some studies with extreme effect sizes (outliers) might cause concerns and distort our pooled effect^45^. So, the influence analyses were performed to detect and remove outliers amongst all eligible studies by using the R function of *influence.analysis*. The publication bias was examined using the *Begg’s* rank correlation^46^ and *Egger’s* weighted regression tests^47^, visualised by funnel plots with based on “trim and fill” test^48^. The sensitivity of the results was examined using the *leave1out* function. Permutation tests, which assumed normality of the observed effects and relied on the asymptotic behaviour of the test statistics, were also performed using the *permutest* function with 1000 iterations to control the Type I error rate. All significance tests were two-tailed, and the significance threshold was set to < 0.05.

## Results

### Characteristics of eligible studies and samples

A total of 87 studies met the inclusion criteria. Figure 1 shows the flowchart of literature search. As described in the Supplementary File, 125 samples and 60,262 subjects were involved. Approximately 45.26% of the subjects were women. About 45.71% (samples = 84, *n* = 27,548) and 50.35% (samples = 37, *n* = 30,340) of the subjects were Asians and Caucasians, respectively. One sample (*n* = 128) was from Africa^49^, and three samples (3.94%, *n* = 2246) were mixed population^50,51^. According to the participants’ clinical information, 52 patient samples (28.04%, *n* = 16,900), 47 random samples (53.94%, *n* = 32,504; including 21 general population and 22 control samples of case–control studies) and mixed samples (i.e. including patients and controls; 18.02%, *n* = 10,858) were obtained from all studies. More than 12 kinds of diseases, including coronary artery disease (samples = 13), coronary artery disease (samples = 8), dyslipidaemia (samples = 7), type 2 diabetes (samples = 6), overweight (samples = 6), Alzheimer’s disease (samples = 2), acute myocardial infarction (samples = 2), Parkinson’s disease, cerebral infarction, frontotemporal dementia, abdominal aortic aneurysm, ischemic heart disease and preeclampsia, were included in these eligible studies. Amongst 125 samples, the distribution of R219K genotypes in seven samples^13,52–57^ was reported to significantly deviate from the HWE, and another seven samples (Chen et al.^58^; hypercholesterolemic group of Katerina et al.^52^; Liu et al.^59^; Wang et al.^60^; PD patients of Ya and Lu^61^; and female group of Zhao et al.^62^; Supplementary File) in which the distribution of R219K genotypes were also not in HWE assessed with R package of *HardyWeinberg*^36^.

### Relationship between R219K and HDLC levels

#### Overall pooled effect of R219K on the HDLC level

Table 1 shows that regardless of model (i.e. in three codominant models, dominant and recessive genetic models), the R219K polymorphism had a solid effect on the human HDLC level (all *P*s < 0.01). This consistent result was observed after the detection and removal of outliers (all *P*s < 0.01). For instance, under the recessive genetic model, the homozygous R allele had a significantly lower HDLC level than the K allele carrier (SMD = − 0.25 mmol/L, 95%CI − 0.32 to − 0.18, *z* = − 6.96, *P* < 0.01), as observed using the random-effects model. Extreme heterogeneity (*I*^2^ = 93.31%, *Q* = 1763.65, df = 118, *P* < 0.01) was also observed amongst eligible studies. A consistent relationship was observed after running 1000 iterations for the approximate permutation test (*P*_permutation_ = 0.01). Amongst the eligible studies, six samples (EI-Aziz et al.^17^, data 1 of Çoban et al.^63^, two samples of Sun et al.^64^, and Ya et al.’s^61^ AD and PD samples) were identified as outliers with low-quality data and extreme effect size (Supplementary Fig. S1) and ruled out in the following analyses. The RR genotype population still showed a significantly lower HDLC level (SMD = − 0.13 mmol/L, 95%CI − 0.17 to − 0.08, *z* = − 6.11, *P* < 0.01, *P*_mutation_ < 0.01; and *I*^2^ = 80.42%, *Q* = 572.04, df = 112, *P* < 0.01) than K allele carriers in the random-effects model.

**Table 1:** Meta-analysis of the association of *ABCA1* R219K polymorphism and lipid profiles.

Genetic model	Test of association				Test of heterogeneity			Publication bias			
	*SMD*	95% CI	*z*	*P*	*tau*^2^	*P*	*I*^2^ (%)	*Egger*’s test		*Begg*’s test	
								*z*	*P*	*tau*	*P*
**HDLC**											
Codominant 1 (RK vs. KK)	− 0.33	− 0.45 to − 0.20	− 5.20	** < 0.01**	0.35	** < 0.01**	94.90	− 2.99	** < 0.01**	− 0.20	** < 0.01**
Outliers removed	− 0.18	− 0.24 to − 0.11	− 5.05	** < 0.01**	0.08	** < 0.01**	81.62	− 2.29	** 0.02**	− 0.12	0.08
Codominant 2 (RR vs. KK)	− 0.49	− 0.63 to − 0.38	− 6.81	** < 0.01**	0.46	** < 0.01**	95.62	− 6.83	** < 0.01**	− 0.28	** < 0.01**
Outliers removed	− 0.22	− 0.29 to − 0.15	− 6.25	** < 0.01**	0.08	** < 0.01**	80.00	− 3.75	** < 0.01**	− 0.17	** 0.01**
Codominant 3 (RK vs. RR)	0.21	0.13 to 0.30	4.89	** < 0.01**	0.16	** < 0.01**	94.51	3.55	** < 0.01**	0.28	** < 0.01**
Outliers removed	0.09	0.04 to 0.14	3.71	** < 0.01**	0.04	** < 0.01**	81.30	2.07	** 0.04**	0.20	** < 0.01**
Dominant model (RR + RK vs. KK)	− 0.30	− 0.44 to − 0.16	− 4.19	** < 0.01**	0.49	** < 0.01**	96.69	− 1.28	0.20	− 0.17	** 0.01**
Outliers removed	− 0.23	− 0.30 to − 0.15	− 5.83	** < 0.01**	0.12	** < 0.01**	87.66	− 2.66	** < 0.01**	− 0.15	** 0.03**
Recessive model (RR vs. RK + KK)	− 0.25	− 0.32 to − 0.18	− 6.96	** < 0.01**	0.13	** < 0.01**	93.31	− 4.36	** < 0.01**	− 0.28	** < 0.01**
Outliers removed	− 0.13	− 0.17 to − 0.08	− 6.11	** < 0.01**	0.04	** < 0.01**	80.42	− 3.01	** < 0.01**	− 0.22	** < 0.01**
Over dominant model (RK vs. RR + KK)	0.00	− 0.03 to 0.04	0.20	0.84	0.02	** < 0.01**	65.10	1.51	0.13	0.07	0.32
Outliers removed	− 0.00	− 0.03 to 0.02	− 0.19	0.85	0.01	** < 0.01**	39.48	0.38	0.70	0.02	0.75
**LDLC**											
Codominant 1 (RK vs. KK)	− 0.08	− 0.16 to − 0.00	− 2.03	** 0.04**	0.09	** < 0.01**	79.32	− 0.39	0.69	0.03	0.72
Outliers removed	− 0.04	− 0.09 to 0.01	− 1.48	0.14	0.03	** < 0.01**	55.20	2.09	** 0.04**	0.09	0.24
Codominant 2 (RR vs. KK)	− 0.04	− 0.13 to 0.05	− 0.81	0.42	0.13	** < 0.01**	82.80	− 0.61	0.54	0.09	0.25
Outliers removed	0.01	− 0.06 to 0.08	0.30	0.76	0.06	** < 0.01**	68.65	2.11	** 0.04**	0.14	0.06
Codominant 3 (RK vs. RR)	− 0.03	− 0.12 to 0.05	− 0.72	0.47	0.13	** < 0.01**	91.43	− 0.35	0.73	0.06	0.44
Outliers removed	− 0.05	− 0.10 to − 0.00	− 2.05	** 0.04**	0.03	** < 0.01**	67.54	0.18	0.86	− 0.08	0.31
Dominant model (RR + RK vs. KK)	− 0.22	− 0.39 to − 0.06	− 2.63	** < 0.01**	0.55	** < 0.01**	96.15	15.21	** < 0.01**	− 0.01	0.90
Outliers removed	− 0.03	− 0.14 to 0.08	− 0.53	0.59	0.22	** < 0.01**	91.06	15.74	** < 0.01**	0.13	0.08
Recessive model (RR vs. RK + KK)	0.04	− 0.01 to 0.10	1.65	0.10	0.05	** < 0.01**	81.29	1.25	0.21	0.11	0.11
Outliers removed	0.02	− 0.02 to 0.06	1.11	0.27	0.02	** < 0.01**	63.35	1.44	0.15	0.10	0.16
Over dominant model (RK vs. RR + KK)	− 0.05	− 0.09 to − 0.00	− 2.12	** 0.03**	0.02	** < 0.01**	69.10	1.02	0.31	0.01	0.91
Outliers removed	− 0.02	− 0.04 to 0.01	− 1.21	0.22	0.00	** 0.03**	24.16	0.41	0.69	0.04	0.56
**TC**											
Codominant 1 (RK vs. KK)	− 0.04	− 0.22 to 0.14	− 0.45	0.66	0.65	** < 0.01**	96.20	0.43	0.67	0.07	0.36
Outliers removed	0.03	− 0.04 to 0.11	0.92	0.36	0.08	** < 0.01**	74.75	1.15	0.25	0.18	** 0.02**
Codominant 2 (RR vs. KK)	− 0.04	− 0.25 to 0.16	− 0.40	0.69	0.89	** < 0.01**	96.92	0.62	0.54	0.05	0.52
Outliers removed	0.02	− 0.06 to 0.10	0.43	0.66	0.10	** < 0.01**	77.27	1.64	0.10	0.14	0.08
Codominant 3 (RK vs. RR)	0.01	− 0.07 to 0.08	0.23	0.82	0.10	** < 0.01**	88.93	− 0.54	0.59	0.11	0.12
Outliers removed	− 0.00	− 0.06 to 0.05	− 0.12	0.91	0.05	** < 0.01**	77.97	0.00	0.99	0.02	0.78
Dominant model (RR + RK vs. KK)	− 0.11	− 0.32 to 0.11	− 0.96	0.34	1.03	** < 0.01**	97.85	0.28	0.78	− 0.00	0.96
Outliers removed	0.01	− 0.08 to 0.10	0.20	0.84	0.13	** < 0.01**	85.09	1.14	0.25	0.14	0.07
Recessive model (RR vs. RK + KK)	− 0.03	− 0.11 to 0.05	− 0.79	0.43	0.15	** < 0.01**	92.39	− 1.91	0.06	− 0.11	0.09
Outliers removed	0.02	− 0.02 to 0.06	0.96	0.34	0.03	** < 0.01**	67.13	0.97	0.33	0.02	0.77
Over dominant model (RK vs. RR + KK)	0.01	− 0.05 to 0.06	0.21	0.83	0.05	** < 0.01**	82.59	0.85	0.39	0.10	0.18
Outliers removed	− 0.01	− 0.03 to 0.05	− 0.37	0.71	0.02	** < 0.01**	66.26	0.80	0.42	0.11	0.16
**TG**											
Codominant 1 (RK vs. KK)	0.06	− 0.10 to 0.21	0.73	0.47	0.48	** < 0.01**	95.06	− 2.77	** < 0.01**	0.06	0.39
Outliers removed	0.18	0.09 to 0.26	4.01	** < 0.01**	0.12	** < 0.01**	83.14	0.30	0.77	0.17	** 0.03**
Codominant 2 (RR vs. KK)	0.20	− 0.01 to 0.41	1.89	0.06	0.88	** < 0.01**	96.88	− 3.52	** < 0.01**	0.04	0.58
Outliers removed	0.16	0.08 to 0.25	3.67	** < 0.01**	0.11	** < 0.01**	80.09	0.58	0.56	0.09	0.23
Codominant 3 (RK vs. RR)	− 0.10	− 0.24 to 0.04	− 1.45	0.15	0.37	** < 0.01**	96.60	1.48	0.14	− 0.08	0.31
Outliers removed	− 0.03	− 0.09 to 0.03	− 0.93	0.35	0.06	** < 0.01**	81.58	0.98	0.33	− 0.01	0.92
Dominant model (RR + RK vs. KK)	0.12	− 0.05 to 0.29	1.36	0.17	0.60	** < 0.01**	96.45	0.70	0.48	0.04	0.56
Outliers removed	0.17	0.07 to 0.27	3.24	** < 0.01**	0.19	** < 0.01**	89.29	0.06	0.95	0.14	0.06
Recessive model (RR vs. RK + KK)	0.18	0.06 to 0.30	3.01	** < 0.01**	0.35	** < 0.01**	96.38	7.39	** < 0.01**	0.12	0.07
Outliers removed	0.07	0.02 to 0.12	2.25	** 0.01**	0.06	** < 0.01**	82.03	− 0.43	0.67	0.04	0.62
Over dominant model (RK vs. RR + KK)	− 0.02	− 0.09 to 0.06	− 0.40	0.69	0.11	** < 0.01**	91.43	0.78	0.44	0.01	0.86
Outliers removed	− 0.02	− 0.07 to 0.03	− 0.68	0.50	0.03	** < 0.01**	76.61	2.06	0.04	0.02	0.76

SMD: standard mean difference; CI: confidence interval.

Bold indicates statistically significant (*P* < 0.05).

#### Effect of R219K on the HDLC levels of Asian and Caucasian subgroups

Hierarchical meta-analyses were performed to estimate the influence of race on the R219K effect (Supplementary Table S3). Consistent with the results of total samples, significant effects of R219K on the human HDLC level in the Asian population (all *P*s < 0.01) with extremely heterogeneity (all *I*^2^ > 56% and *P*s < 0.01) were observed under codominant, dominant and recessive genetic models. Under difference genetic models, Caucasian populations did not show a consistent result (Supplementary Table S3). The difference of the R219K effects on the HDLC level between Asian and Caucasian populations was estimated, showing a significant difference under the codominant 2 model (RR vs. KK, SMD = − 0.23 mmol/L, 95%CI − 0.37 to − 0.21 vs. SMD = − 0.05 mmol/L, 95%CI − 0.18 to 0.08; and *Q* = 9.71, df = 1, *P* < 0.01) and a weak difference under the recessive model (RR vs. RK + KK, SMD = − 0.17 mmol/L, 95%CI − 0.22 to − 0.12 vs. SMD = − 0.08 mmol/L, 95%CI − 0.16 to 0.01; and *Q* = 3.58, df = 1, *P* = 0.06). No significant difference was observed under other genetic models (Supplementary Table S3).

#### Effect of R219K on the HDLC levels in different health status subgroups

Supplementary Table S3 and Supplementary Fig. S2 show the difference of effect of R219K on the HDLC level by considering participants’ clinical information. Under two codominant, dominant and recessive models, a significant correlation was observed between R219K and HDLC level regardless of patient group (all *P*s < 0.02), random population (general, all *P*s < 0.02) and mixed population (all *P*s < 0.01). For instance, under the recessive genetic model, the average HDLC level in the RR genotype group was significantly lower than that in K allele carriers within patient samples (SMD = − 0.15 mmol/L, 95%CI − 0.23 to − 0.06, *z* = − 3.46,* P* < 0.01), and the same trend was also observed in random and mixed populations (random population: SMD = − 0.11 mmol/L, 95%CI − 0.17 to − 0.05, *z* = − 3.60, *P* < 0.01; mixed population: SMD = − 0.14 mmol/L, 95%CI − 0.24 to − 0.05, *z* = − 2.98,* P* < 0.01). Additionally, under the codominant 1 (RK vs. KK) and dominant (RR + RK vs. KK) models, the effect of R219K was significantly different in accordance with the clinical conditions of different participants (codominant 1 model: *Q* = 8.39, df = 2, *P* = 0.02; dominant model: *Q* = 6.81, df = 2, *P* = 0.03; Supplementary Table S3).

#### Sources of heterogeneity

Moderators, including categorical (e.g. race and health status) and numerical (e.g. publication time, sample size, BMI, sex, age and NOS score) variables, which might contribute to such observed extreme heterogeneity, were analysed using the meta-regression analysis. The influence of each variable on the effect of R219K was estimated. Table 2 describes that the participants’ health status showed an obvious influence on the effect of R219K (*Q* = 4.91, df = 2, *P* = 0.08). Comparing to mix health status participants, the effect of R219K in patients was higher than that of general population (patients: *β* = 0.22, z = 1.63, *P* = 0.10; random samples: *β* = − 0.01, *z* = − 0.10, *P* = 0.92) under the recessive genetic model. Additionally, the sex of participants (i.e. proportion of females in each sample) showed a significant influence on the effect of R219K (*β* = 0.38, *z* = 2.17, *P* = 0.01). Race did not influence the R219K effect, although different relationships were observed in different subgroups (Supplementary Table S3).

**Table 2:** Meta-regression analysis for relationship between R219K and HDLC under recessive model.

Moderator	Studies	*Coefficient (β)*	*SE*	95% CI	*z*	*P*
Intercept		1.56	18.00	− 33.71 to 36.83	0.09	0.93
Publication time	119	< − 0.01	0.01	− 0.02 to 0.02	− 0.02	0.99
Race: Caucasian^a^	35	0.04	0.13	− 0.21 to 0.30	0.34	0.74
Sample size	119	< 0.01	< 0.01	− 0.01 to 0.01	0.85	0.40
**Health condition**^**b**^				*Q* = 4.91, *df* = 2, *P* = 0.08		
Patients	52	0.22	0.13	− 0.04 to 0.48	1.63	0.10
Random	45	− 0.01	0.14	− 0.28 to 0.26	− 0.10	0.92
Sex	113	0.38	0.15	0.08 to 0.67	2.50	**0.01**
Age	115	< − 0.01	< 0.01	− 0.01 to 0.01	− 0.96	0.34
BMI	85	− 0.03	0.02	− 0.08 to 0.02	− 1.14	0.26
NOS	119	− 0.10	0.06	− 0.21 to 0.01	− 1.87	0.06

Test of the model: *Q*_*M*_ = 19.07, *df* = 9, *P* = 0.02; Goodness of fit test: *Q*_*G*_ = 989.09, *df* = 72, *P* < 0.01.

Bold indicates statistically significant (*P* < 0.05).

BMI, body mass index; NOS, Newcastle–Ottawa Scale assessment scale.

^a^Samples of Asian as reference.

^b^Samples including patients and controls (mix) set as reference.

#### Publication bias analysis

Amongst five genetic models, the methods of Begg’s rank correlation and Egger’s weighted regression detected significant publication selection bias in this meta-analysis. For instance, under the recessive model, both of methods suggested significant publication bias consistently (Begg’s test: *tau* = − 0.28, *P* < 0.01; Egger’s test: *z* = − 4.36, *P* < 0.01). The funnel plot also showed a considerable asymmetry distribution amongst the included studies (Supplementary Fig. S3). Furthermore, the trim-and-fill test indicated approximately 34 studies on the left side of the mean effect missing, and a consistent overall effect (*SMD*_adj_ = − 0.42 mmol/L, 95%CI − 0.50 to − 0.34; *z* = − 10.33, *P* < 0.01) was observed after adjustment. The sensitivity analysis indicated that the effect sizes of removing any single study did not deviate from the overall effect.

### Meta-analysis for the genetic variant R219K and LDLC levels

The pooled effect of the genetic variant R219K on LDLC levels was estimated using 74 eligible studies (samples = 104) including 39,323 participants. Under six genetic models, the relationship between R219K and individual’s LDLC level was estimated using the meta-analysis. Under the dominant model, R allele carriers (i.e. with RR + RK genotypes) showed a significantly lower LDLC level than the KK genotype group (SMD = − 0.22 mmol/L, 95%CI − 0.39 to − 0.06, *z* = − 2.63, *P* < 0.01). Under codominant (RK vs. KK genotype) and over dominant (RK vs. RR + KK genotypes) models, a weak correlation between R219K and LDLC level was also detected (codominant: SMD = − 0.08 mmol/L, 95%CI − 0.16 to − 0.03, *z* = − 2.03, *P* = 0.04; over dominant: SMD = − 0.05 mmol/L, 95%CI − 0.09 to − 0.01, *z* = − 2.12, *P* = 0.03). However, no significant correlation was observed under these three genetic models (all *P*s > 0.05, Table 1) after removing three studies with outliers^63,65,66^ (Supplementary Fig. S4).

For potential modulators (e.g. race, BMI, health status, publication time, sample size, gender, age and NOS score), no significant modulation on the effect of R219K was found using hierarchical and meta-regression analyses. The Begg’s rank correlation and Egger’s weighted regression tests showed no consistent publication bias in all genetic models.

### Meta-analysis for the genetic variant R219K and TC levels

The effect size of the genetic variant R219K on TC levels was pooled from 69 studies (samples = 106, *n* = 35,885). The meta-analysis showed no significant difference in the TC levels of the RR genotype population and K allele carriers in the random model and a significant heterogeneity amongst all studies. After removing the five outliers (Çoban et al.^63^ data1; Katzov et al.^67^ and Ya^61^ data1, 2 and 3; Supplementary Fig. S5) detected by the *meta* and *metafor* packages, a consistent result was obtained. Hierarchical and meta-regression analyses, which were used to explore heterogeneity amongst samples, did not observe any moderator impacted the effect of R219K. Furthermore, no significant publication bias was found amongst the current selected studies.

### Meta-analysis for the genetic variant R219K and TG levels

A total of 76 eligible studies (samples = 103, *n* = 38,304) were collected in this study to explore the relationship between the R219K polymorphism and individual TG levels. Under the recessive model, the RR genotype population had significantly higher TG level than K allele carriers (SMD = 0.18 mmol/L, 95%CI 0.06 to 0.30, *z* = 3.01, *P* < 0.01), and *I* ^2^ = 96.38% (*Q* = 2,816, df = 106, *P* < 0.01) for the heterogeneity test. After removing six outliers (Çoban^63^ data 1 and data 2, Delgado-Lista^68^, Sun^64^ data1 and data 2, Ya^61^ data1; Supplementary Fig. S6), the significant effect of R219K was also observed in two codominant (RK vs. KK: SMD = 0.18 mmol/L, 95%CI 0.09 to 0.26, *z* = 4.01, *P* < 0.01; RR vs. KK: SMD = 0.16 mmol/L, 95%CI 0.08 to 0.25, *z* = 3.67, *P* < 0.01), dominant (RR + RK vs. KK: SMD = 0.17 mmol/L, 95%CI 0.07 to 0.27, *z* = 3.24, *P* < 0.01) and recessive (RR vs. KK + RK: SMD = 0.07 mmol/L, 95%CI 0.01 to 0.12, *z* = 2.56, *P* = 0.01) models.

The influences of ethnicity and health status of participants on the effect of R219K were investigated, and a complex relationship was observed. Under four genetic models (i.e. RK vs. KK; RR vs. KK; RR + RK vs. KK; and RR vs. RK + KK), R219K had a significant effect on the TG levels (all *P*s < 0.01, Supplementary Table S4) within Asian populations. For instance, Asians with the RR genotype had a significantly lower TG level than those with K allele carriers (RR vs. RK + KK: SMD = 0.08 mmol/L, 95%CI 0.02 to 0.13, *z* = 2.65, *P* < 0.01). Only a weak effect was observed within the Caucasian population (RR vs. RK + KK: SMD = 0.02 mmol/L, 95%CI − 0.12 to 0.16, *z* = 0.27, *P* = 0.78; Supplementary Fig. S7). Unfortunately, the R219K effects between two populations did not reach a significant difference. Considering the health status of participants (i.e. patients, general population and mixed population), significant effects of R219K on the TG level were observed under two codominant models (RK vs. KK and RR vs. KK; all *P*s < 0.05) in all three subgroups. No significant effect was detected under three genetic models (RK vs. RR; RR vs. RK + KK; and RK vs. RR + KK; all *P*s > 0.05), and an inconsistent result was observed under the dominant model (i.e. RR + RK vs. KK; Supplementary Table S4). In addition, the influence of the sex (i.e. the proportion of females in each sample) on the effect of R219K was observed. The meta-regression analysis revealed that the higher the proportion of females in samples, the higher the effect was detected (*β* = − 1.57, 95%*CI* − 2.20 to − 0.93, *z* = − 4.5, *P* < 0.01). The significant influence of NOS score on relationship between R219K and TG level under recessive model (*β* = 0.27, 95%CI 0.03 to 0.52, *z* = 2.17, *P* = 0.03; Supplementary Table S5) also suggested that the studies quality should be considered. No significant publication bias was found using the Begg’s rank correlation and Egger’s weighted regression methods. The sensitivity analysis showed that effect sizes did not change remarkably after removing any single study.

## Discussion

Given the importance of ABCA1 in the formation of nascent HDLC^69–71^ and its role in the reverse cholesterol transportation^72^, the relationship between the genetic variant R219K and serum lipid levels (i.e. HDLC, LDLC, TG and TC), and the risk of common human diseases (e.g. CVD^23,73^, diabetes^74^ and obesity^49^) has been extensively investigated. Thus, this polymorphism has been observed as a promising prognostic and predictive biomarker of these diseases for susceptible individuals^23^. However, such relationship between this polymorphism and lipid levels still presents some controversies. Here, we conducted four updated meta-analyses based on current studies and observed the consistent significant effect of R219K on the level of HDLC under codominant, dominant and recessive genetic models. At the same time, the weak correlation of R219K with LDLC and TG levels was detected.

HDLC is the well-behaved “good cholesterol” because it removes harmful “bad cholesterol” from the body, and increasing evidence showed that the K allele of R219K is positively associated with elevated HDLC level and lower risks of common human diseases (e.g. CVD, diabetes and stroke)^75^. On the basis of the data of more than 80 studies, this study confirmed the significant effect of R219K on the level of HDLC under the codominant, dominant and recessive genetic models (all *P*s < 0.01) even after the removal of outliers and the correction of permutation test with 1000 iterations. This finding was consistent with previous reports^28,29^. However, the effect of R219K on the levels of TG and LDLC seemed somewhat unclear. For the TG level, R219K showed a consistent effect only in the recessive model (i.e. RR vs. RK + KK; Table 1). Previous studies^63,76,77^ reported that the RR genotype had a higher level of TG than K carriers (SMD = 0.18 mmol/L, 95%CI 0.06 to 0.30, *z* = 3.01, *P* < 0.01). For the LDLC level, some studies^65,78^ and meta-analyses^28^ reported the association between R219K and LDLC level, which disappeared when studies with the outliers were removed. Such results demonstrated the value of R219K in the clinical application of the above mentioned diseases’ diagnosis, monitoring and strategic therapeutic approach design^75^, but other potential elements, such as the influence of outliers, race, gender, and age, should be treated with caution.

The fluctuation of the effect of R219K on lipid levels for each study is becoming another more attractive issue. Subgroup analyses and meta-regression tests indicated that besides the influence of some extreme effect sizes, other factors (including race, gender, age and health status of participants) should be considered in this study. Firstly, populations with different conditions (i.e. patient, random and mixed groups) had significantly different effects of R219K. For instance, significantly different effects of R219K were observed on the HDLC level in groups with different health status (patients: SMD = − 0.15 mmol/L; general population: SMD = − 0.16 mmol/L; mix population: SMD = − 0.49 mmol/L; *Q* = 0.07, df = 2, *P* = 0.03; Supplementary Table S3 and Supplementary Fig. S2) in the dominant genetic model. Secondly, as previous studies^79,80^ mentioned, racial/ethnic difference was observed. Compared with those of Caucasian populations, the effect of R219K on the HDLC and TG levels of Asian populations showed more significance and consistency. For the TG level, the effect of R219K was observed under three genetic models (all *P*s < 0.01, Supplementary Table S4) in the Asian population only. Furthermore, the influence of sex on the effects of R219K on the levels of HDLC and TG was determined using the meta-regression test. Results showed that samples with different proportions of female participants had varying effects of R219K on HDLC and TG levels (Table 2, Supplementary Table S5 and Supplementary Fig. S7). These variables might be the reason for the inconsistency on the relationship between R219K and lipid profiles and for the sources of the extreme heterogeneity observed in this study.

The present study had several strengths. (i) It used a robust, systematic and transparent approach in accordance with the Cochrane Handbook and the PRISMA statement. Compared with previous meta-analyses, this study explored the heterogeneity sources by using larger and more comprehensive samples (samples = 22, *n* = 21,966 in Ma et al.^29^; samples = 62, *n* = 48,452 in Lu et al.^28^, and only type 2 diabetes studies were considered in Jung et al.^22^). (ii) The *metafor* package was used for the detection and removal of outliers from total studies to minimise their influence, which usually had a decreased quality and extreme effect size, and obtain a robust pooled effect. The obvious influence of the study’s quality (i.e. NOS score) on the findings (Tables 1 and S5) also demonstrated the necessity of outlier analysis. (iii) All genetic models were introduced in this study. The genetic association study in practice assumed a specific genetic model, such as dominant or recessive, but conclusions might be sensitive to this assumption^81^. In this study, Table 1 shows the effects of R219K on lipid profiles under six genetic models and demonstrates the sensitivity of this effect.

The limitations of this review must also be mentioned. Firstly, a systemic meta-analysis should collect as much literature as possible, even unpublished studies. However, most of these eligible studies were in English or Chinese. Publication bias analyses also suggested the influence of missing publication studies because the reporting bias^82^ should be considered (e.g. Supplementary Fig. S3). It is possible that such factor can affect the validity and generalization of our findings about the relationship between R219K and HDLC, LDLC, Cholesterol and TG. Secondly, the random-effects model was predominantly adopted to address the extremely significant heterogeneity amongst the total samples. Hierarchical and regression meta-analyses were also performed to explore the source of heterogeneity, but the source of most of the variances in the effect remained unknown. Thirdly, the health status of the subjects was introduced as a categorical variable to explore its influence on R219K effect, and all patients with six kinds of cardiovascular diseases, diabetes and other diseases were classified into one group. However, this “unified” approach might have caused sample heterogeneity in this study. Given sufficient eligible studies for each disease, a network meta-analysis should be performed to determine the comparative effects of all included diseases^83^.

## Conclusion

The present meta-analyses confirmed the effect of R219K in the *ABCA*1 gene on the level of lipids. Individuals with different genotypes have different levels of lipids (HDLC and TG), which may result in different risks of human diseases. The influences of ethnicity and health status on pooled effects must be considered when interpreting current findings and/or accepting the recommendation for R219K clinical applications in the future.

## Supplementary Information

Supplementary Information.

## Data Availability

Data used for this study are available from the authors of each included study upon reasonable request. All data generated or analysed during this study are included in this article and its Online Resources.
