# Supplementary material for: Association between the ABCA1 (R219K) polymorphism and lipid profiles: a meta-analysis
Source: Sci Rep. 2021 Nov 5;11:21718. doi: 10.1038/s41598-021-00961-9 (PMC8571387; doi:10.1038/s41598-021-00961-9)
Supplement: Supplementary file 1 — Supplementary Information. [file 41598_2021_961_MOESM1_ESM.pdf]

Supplementary file

Association between the ABCA1 (R219K) polymorphism and lipid profiles: A meta-analysis

Zhangyan Shi [1], Yajie Tian [1], Ze Zhao [1], Yufei Wu [1], Xiuxia Hu [1], Junlin Li [1,2], Qianliang Chen[3], Yan Wang [1], Caiyan An[4 *], Kejin Zhang[1,2,5 *]

[1] College of Life Science, Northwest University, Xi'an, 710069, China.

[2] College of Medicine, Northwest University, Xi'an 710069, China.

[3] Shaanxi Key Laboratory of biomedicine, College of Life Science, Northwest University, Xi'an 710069, China.

[4] School of Basic Medical Science, Inner Mongolia Medical College, Hohhot 010059, China.

[5] Institute of Population and Health, Northwest University, Xi'an 710069, China.

[*]Corresponding author:

Kejin Zhang: Institute of Population and Health, College of Life Science, Northwest University, Xi'an 710069, China. zhangkj@nwu.edu.cn. ORCID: 0000-0002-4980-1736

Caiyan An: School of Basic Medical Science, Inner Mongolia Medical College, Hohhot 010059, China. acy_1999@163.com

1 METHODS AND MATERIALS

1.1 Full search strategy in data bases

Boolean/Phrase:

1.1.1 For Web of Science, MEDLINE, BIOSIS, EMBASE, Scopus, Derwent Innovations Index, InspecR, KCI-Korean Journal Database, and SciELO Citation Index data bases

TS = (("adenosine triphosphate - binding cassette transporter A1" OR "ATP-binding cassette transporter A1" OR ABCA1 OR rs2230806 OR R219K OR R1587K OR G1051A)) AND TS = ((lipid OR triglyceride OR cholesterol))

1.1.2 For PubMed data base

(((((((adenosine triphosphate - binding cassette transporter A1[Title/Abstract]) OR ATP-binding cassette transporter A1[Title/Abstract]) OR ABCA1[Title/Abstract]) OR rs2230806[Title/Abstract]) OR R219K[Title/Abstract]) OR R1587K[Title/Abstract]) OR G1051A[Title/Abstract]) AND (((lipid[Title/Abstract]) OR triglyceride[Title/Abstract]) OR cholesterol[Title/Abstract])

1.1.3 For CNKI data base

AB = (‘adenosine triphosphate - binding cassette transporter A1’ + ‘ATP-binding cassette transporter A1’ + ‘ABCA1’ + ‘rs2230806’ + ‘R219K’ + ‘R1587K’ + ‘G1051A’) and (AB = ‘lipid’ + ‘triglyceride’ + ‘cholesterol’)

1.1.4 For Wanfang data base

(题名或关键词: “adenosine triphosphate - binding cassette transporter A1” + “ATP-binding cassette transporter A1” + “ABCA1” + “rs2230806” + “R219K” + “R1587K” + “G1051A”) and (题名或关键词: “lipid” + “triglyceride” + “cholesterol”)

1.2 Converting and formatting of data sets

1.2.1 Convert the units of lipid levels into the same unit (mmol/L)

Among these eligible studies, two units (the unit of mmol/L were used in eighteen studies[1-18]; and the unit of mg/dL in the rest studies; Supplementary Table S1 and S2) were mainly used to describe one’s lipid levels. The unit of lipid-levels data (e.g., mean, SD or SE) had been converted to mmol/L in this study, according to Rugge B’s formulas [19]:

To Convert From mg/dL to mmol/L

For total, HDL, and LDL cholesterol divided mg/dL by 38.67

e.g., 135 mg/dL = 3.5 mmol/L * 38.67 = 3.5 mmol/L

For trglycerides divided mg/dL by 88.57

e.g., 168 mg/dL = 1.9 mmol/L * 88.57 = 1.9 mmol/L

(Formulas 1/3)

The lipid profiles in eight studies [6-10,12,16,20](sample n = 14) were described with median and interquartile range (IQR) values. The mean and SD values of them will be converted by another online program [21] (http://vassarstats.net/median_range.html). (Table S2)

1.2.2 Combining two groups mean, sd and sample size

To get the data of three combining groups (RR + RK, KK + RK and RR + KK), a combination of two sets data (including mean, sd and sample size) was performed with an online program (https://www.statstodo.com/CombineMeansSDs_Pgm.php). Within this program, the data for each two genotypes (e.g., RK and KK) were used to recreate a new data set for K allele carrier group, employing the following formula:

The combined mean is computed as the weighted mean (by sample size) across groups,

$$\bar{X}_1 = \frac{n_{11}\bar{X}_{11} + n_{12}\bar{X}_{12}}{n_{11} + n_{12}}$$

(Formulas 2/3)

and the combined standard deviation is computed as

$$S_1 = \sqrt{\frac{(n_{11}-1)S_{11}^2 + (n_{12}-1)S_{12}^2 + \frac{n_{11}n_{12}}{n_{11}+n_{12}}(\bar{X}_{11} + \bar{X}_{12})^2}{n_{11}+n_{12}-1}}$$

(Formulas 3/3)

where$\bar{X}_{11}$, $\bar{X}_{12}$ are the means in subgroups 1 and 2 of treatment group; S_{11}^2, $S_{12}^2$the standard deviations, and n_{11}, $n_{11}$the sample sizes of subgroups 1 and 2.

1.3 Hardy-Weinberg equilibrium (HWE) test for R219K genotype distribution

In each samples of studies, the information about the deviation of genotypes from HWE were also collected as one of quality evaluation. A R package of Hardy-Weinberg [22] was used to estimate the deviation of genotypes from HWE in samples, given the information about HWE test unavailable.

1.4 Extracted data for meta-analysis

Supplementary Table S1 listed the demographic information, health status, assessment of quality (Newcastle-Ottawa Scale, NOS) and Hardy-Weinberg equilibrium (HWE) test of each eligible study. In supplementary Table S2, the lipid profiles of different genotypes individuals within each study was described.

1.4.1 The demographic information, health status, assessment of study quality and HWE test of all studies

No.	Authors	NOS1	NOS2	HWE	Country	Race	Size	Diseases	Healthy	Sex	Age	BMI
1	Abd, 2014	8	7	Y	Egypt	Caucasian	235	mix	mix	0.35	42.15	28.9
2	Abellan, 2010	6	6	Y	Spain	Caucasian	1,473	random	random	0.50	52.70	26.3
2	Abellan, 2010	6	6	Y	Spain	Caucasian	627	random	random	1.00	52.70	25.7
3	Akao, 2014	6	6	Y	USA	Caucasian	2,619	random	random	0.00	75.30	26.8
3	Akao, 2014	6	6	Y	USA	Caucasian	2,795	random	random	1.00	75.30	26.8
4	Balcerzyk, 2007	7	7	Y	Poland	Caucasian	178	CAD	patient	0.34	43.81	25.9
5	Benton, 2007	6	7	Y	mixed	White/Chinese/Hispanic	6,814	random	random	0.45	62	28.3
6	Cai, 2017	8	7	Y	China	Asian	124	T2DM	patient	0.51	62.46	25.1
6	Cai, 2017	8	7	Y	China	Asian	102	CTR	random	0.45	60.52	25
7	Cenarro, 2003	9	8	Y	Spain	Caucasian	210	mix	mix	0.00	55.25	27.1
7	Cenarro, 2003	9	8	Y	Spain	Caucasian	164	mix	mix	1.00	64.92	27.5
8	Chen, 2009	6	7	YN	China	Asian	500	AF	patient	0.40	64.05	23.5
9	Clee, 2001	6	6	N	Canada	Caucasian	790	CAD	patient	0.00	56.16	26
10	Coban, 2014	9	8	Y	Turkey	Caucasian	267	mix	mix	0.00	58.90	27.7
10	Coban, 2014	9	8	Y	Turkey	Caucasian	330	mix	mix	1.00	56.70	30.6
11	Delgado-Lista, 2010	7	6	Y	Spain	Caucasian	88	health	random	0.00		
12	Deng, 2008	7	7	Y	China	Asian	448	mix	mix	0.43	61.94	23.5
13	Du, 2020	8	8	Y	China	Asian	996	T2DM	patient	0.50	60.21	26.44
14	Evans, 2003	8	8	Y	Germany	Caucasian	515	CHD	patient		45.59	26.3
15	Genvigir, 2008	6	6	N	Brazil	Caucasian	224	hypercholesterolemic	patient	0.67	55.00	27
15	Genvigir, 2008	6	6	N	Brazil	Caucasian	143	CTR	random	0.73	47.00	26
16	Ghaznavi, 2018	7	7	Y	Iran	Caucasian	100	CAD	patient	0.50	58.96	
16	Ghaznavi, 2018	7	7	Y	Iran	Caucasian	100	CTR	random	0.50	57.53	
17	Guo, 2010	7	7	Y	China	Asian	144	T2DM	patient	0.47	57.49	
18	Haghvirdizadeh, 2015	7	6	Y	Malaysia	Asian	164	T2DM	patient	0.37	62.14	27.9
18	Haghvirdizadeh, 2015	7	6	Y	Malaysia	Asian	165	CTR	random	0.47	54.97	27.1
19	Harada, 2003	8	7	Y	Japan	Asian	265	hypolipidemic	patient	0.13	65.08	23.8
20	Hodoğlugil, 2005	9	8	Y	Turkey	Caucasian	1,551	health	random	0.00	41.00	26.1
20	Hodoğlugil, 2005	9	8	Y	Turkey	Caucasian	1,149	health	random	1.00	41.00	25.7
21	Huang, 2007	8	8	Y	China	Asian	60	CAD	patient	0.00	64.57	
21	Huang, 2007	8	8	Y	China	Asian	53	CAD	patient	1.00	64.57	
22	Huang, 2011	9	8	Y	China	Asian	206	overweight	patient	0.38	55.31	25.8
22	Huang, 2011	9	8	Y	China	Asian	132	CTR	random	0.47	51.70	20.5

23	Katerina, 2010	6	6	N	Czech Republic	Caucasian	340	hypercholesterolemic	random	0.00	47.00	28.4
23	Katerina, 2010	6	6	N	Czech Republic	Caucasian	403	hypercholesterolemic	random	1.00	47.90	25.9
24	Katzov, 2006	9	8	Y	Sweden	Caucasian	2,544	AD, FTD, CTR	mix	0.31	59.60	
25	Kitjaroentham, 2007	8	8	Y	Thailand	Asian	40	overweight	patient	0.00	43.00	29.3
25	Kitjaroentham, 2007	8	8	Y	Thailand	Asian	72	overweight	patient	1.00	43.00	29.3
25	Kitjaroentham, 2007	8	8	Y	Thailand	Asian	96	CTR	random	0.00	37.00	27.8
25	Kitjaroentham, 2007	8	8	Y	Thailand	Asian	21	CTR	random	1.00	37.00	27.8
26	Kolovou F, 2011	8	8	Y	Greece	Caucasian	308	health	random	1.00	22.50	21.5
27	Kolovou, 2012	7	7	N	Greece	Caucasian	447	health	random	0.81	22.00	22.6
28	Kolovou V, 2011	9	8	Y	Greece	Caucasian	586	hypercholesterolemic	patient	0.31	57.20	27
29	Kolovou, 2016	7	7	Y	Greece	Caucasian	432	health	random	0.80	22.00	27.6
30	Kyriakou, 2007	6	6	Y	UK	Caucasian	251	CAD	patient	0.24	59.77	27.5
31	Li, 2005	8	8	Y	China	Asian	396	CHD	patient	0.40	60.10	
32	Li, 2009	9	8	Y	China	Asian	365	CHD	patient	0.52	63.00	
33	Li, 2012	8	8	Y	China	Asian	150	AMI	patient	0.36	62.00	
34	Liu, 2008	7	7	YN	China	Asian	71	T2DM, CHD	patient	0.48	58.00	25
35	Lu, 2014	7	7	Y	China	Asian	999	T2DM	patient	0.50	60.00	25.2
36	Lu, 2020	8	8	Y	China	Asian	217	CTR	random	0.45	61.21	24.2
36	Lu, 2020	8	8	Y	China	Asian	442	CAD	patient	0.36	64.60	23.9
37	Manresa, 2006	7	7	N	Spain	Caucasian	1,248	CHD	random	0.48	51.00	26.5
38	Mantaring, 2007	7	6	Y	Spain	Caucasian	124	mix	patient		52.82	
39	Mao, 2013	7	7	N	China	Asian	199	CHD	patient		54.92	
39	Mao, 2013	7	7	N	China	Asian	83	CTR	random		50.62	
39	Mao, 2013	7	7	N	China	Asian	178	CHD	patient		69.82	
39	Mao, 2013	7	7	N	China	Asian	95	CTR	random		56.94	
40	Marvaki, 2014	6	6	Y	Greece	Caucasian	448	health	random	0.81	22.50	22.6
41	Min, 2014	9	8	Y	China	Asian	305	preeclampsia	patient	1.00		28.2
42	Mokuno, 2015	8	7	Y	Japan	Asian	1,458	mix	random	0.00	56.63	21.7
42	Mokuno, 2015	8	7	Y	Japan	Asian	3,675	mix	random	1.00	56.63	21.7
43	Porchay, 2006	8	8	Y	France	Caucasian	5,040	health	random	0.51	46.80	24.7
44	Porchay, 2009	7	6	Y	France	Caucasian	3,129	T2DM	patient	0.27	65.63	29.4
45	Saleheen, 2007	7	6	Y	Pakistan	Asian	200	hypercholesterolemic	patient	0.36	49.35	
46	Sandhofer, 2008	8	8	Y	Asutria	Caucasian	688	health	random	0.00	49.84	27.2
47	Shi, 2009	8	8	Y	China	Asian	132	CDH	patient	0.36	61.72	24.9
47	Shi, 2009	8	8	Y	China	Asian	157	CTR	random	0.36	61.13	24.2
48	Smirnov, 2018	8	8	Y	Russia	Caucasian	358	dyslipidemia	patient	0.57	48.70	

49	Srinivasan,2003	6	7	Y	mixed	White/black	1,277	health	random		29.75	26.4
50	Sun, 2005	7	7	Y	China	Asian	248	health	random	0.45	59.61	24.2
51	Sun, 2011	9	8	Y	China	Asian	468	health	random	0.28	53.78	26.3
51	Sun, 2011	9	8	Y	China	Asian	640	health	random	0.23	54.77	25.6
52	Takagi, 2002	6	6	Y	Japan	Asian	465	IHD	patient	0.18	58.00	24
53	Teixeira, 2020	8	8	N	Brazil	Caucasian	205	overweight	patient	1.00		33.9
54	Wang X, 2006	9	8	Y	China	Asian	232	CHD	patient	0.39	64 .85	25
55	Wang Y, 2006	8	8	Y	China	Asian	396	CHD	patient	0.40	60.10	
56	Wang, 2004	9	8	Y	China	Asian	222	CHD	patient	0.45	62.37	25.3
56	Wang, 2004	9	8	Y	China	Asian	278	CTR	random	0.49	62.20	24.5
57	Wang, 2008	8	8	Y	China	Asian	93	T2DM	patient	0.30	52.09	25.1
58	Wang, 2009	6	7	N	China	Asian	289	mix	mix	0.27	52.35	
59	Wang, 2010	7	7	Y	China	Asian	476	mix	mix	0.34	65.58	24.1
60	Wang, 2012	9	8	Y	China	Asian	141	CHD	patient	0.32	62.76	24.1
61	Wang, 2013	8	8	Y	China	Asian	114	health	random	0.41	63.81	
62	Wang, 2016	6	6	YN	China	Asian	289	health	random	0.29	41.00	
63	Wang, 2018	7	7	Y	China	Asian	189	health	random	0.32	41.23	
64	Wang, 2021	9	8	Y	China	Asian	145	CTR	random	1.00	30.50	27.4
64	Wang, 2021	9	8	Y	China	Asian	205	preeclampsia	patient	1.00	31.20	30.4
65	Woll, 2005	8	7	Y	USA	Caucasian	838	CAD	patient	0.50	49.50	
65	Woll, 2005	8	7	Y	USA	Caucasian	257	CTR	random	0.50	48.30	
66	Wu, 2006	6	7	Y	China	Asian	87	CAD	mix	0.00		
67	Wu, 2007	9	8	Y	China	Asian	200	CTR	random	0.48	50.57	22.95
67	Wu, 2007	9	8	Y	China	Asian	109	HTG	patient	0.28	52.28	25.05
68	Xia, 2011	7	8	Y	China	Asian	227	CHD	patient	0.52	61.10	25
68	Xia, 2011	7	8	Y	China	Asian	162	CTR	random	0.45	59.50	22.2
69	Xiao, 2004	8	9	Y	China	Asian	730	mix	mix	0.42	62.17	23.6
70	Xiao, 2005	6	7	Y	China	Asian	1,019	mix	mix	0.41	61.73	23.6
71	Xiao, 2012	7	7	Y	China	Asian	208	mix	mix	0.46	77.15	
72	Xue, 2012	9	8	Y	China	Asian	411	mix	mix	0.33	64.98	
73	Ya, 2017	9	8	Y	China	Asian	105	AD	patient	0.43	69.94	
73	Ya, 2017	9	8	Y	China	Asian	116	PD	patient	0.41	68.94	
73	Ya, 2017	9	8	Y	China	Asian	100	CTR	random	0.50	64.85	
74	Yamakawa, 2004	8	7	Y	Japan	Asian	327	health	random	0.50	12.30	19.5
75	Yang, 2011	9	8	Y	China	Asian	266	cerebral infarction	patient	0.37	61.85	24.7
76	Yao, 2016a	8	8	Y	China	Asian	207	CTR	random	0.53	41.04	24

76	Yao, 2016a	8	8	Y	China	Asian	204	obesity	patient	0.47	41.92	25.9
77	Yao, 2016b	8	8	Y	China	Asian	259	overweight	patient	0.44	43.95	27.1
77	Yao, 2016b	8	8	Y	China	Asian	276	CTR	random	0.55	44.32	20.8
78	Yao, 2018	7	8	Y	China	Asian	454	mix	mix	0.49	68.49	23.4
79	Young-Guk, 2003	8	8	Y	Korea	Asian	150	CAD	patient	0.00	56.53	24.9
79	Young-Guk, 2003	8	8	Y	Korea	Asian	403	CTR	random	0.00	43.16	23.7
79	Young-Guk, 2003	8	8	Y	Korea	Asian	48	CAD	patient	1.00	61.12	24.8
79	Young-Guk, 2003	8	8	Y	Korea	Asian	533	CTR	random	1.00	44.48	22.8
80	Yu, 2008	8	8	Y	China	Asian	49	AMI	patient	0.00	55.80	
81	Yuan, 2017	9	9	Y	China	Asian	212	mix	mix	0.52	61.08	
81	Yuan, 2017	9	9	Y	China	Asian	203	mix	mix	0.42	58.42	
82	Zhang L, 2008	8	8	Y	China	Asian	411	mix	mix	0.52	58.76	
83	Zhang X, 2008	9	8	Y	China	Asian	186	CTR	random	0.34	63.50	23.74
84	Zhao, 2004a	8	9	Y	China	Asian	487	mix	mix	0.42	62.00	23.38
85	Zhao, 2004b	7	7	YN	China	Asian	421	mix	mix	1.00	62.00	23.42
85	Zhao, 2004b	7	7	YN	China	Asian	622	mix	mix	0.00	62.00	23.52
86	Zhao, 2016	8	8	Y	China	Asian	126	AAA	patient	0.39	61.50	24.9
87	Zhou, 2013	8	8	Y	China	Asian	630	mix	mix	0.42	62.05	23.61

Supplementary Table S1. The demographic characteristics data of all eligible studies. Abbreviation: AAA, abdominal aortic aneurysm; AD, Alzheimer's disease; AF, atrial fibrillation; AMI, acute myocardial infarction; CAD, coronary artery disease; CHD, coronary heart disease; CTR, control; FTD, frontotemporal dementia; HTG, hypertriglyceridemia; IHD, ischemic heart diseases; PD, Parkinson; T2DM, type 2 diabetes; HWE, Hardy-Weinberg equilibrium test, of which the Y means distribution of genotypes was in HWE, the N was not in HWE, and the YN indicated inconsistent with the original description that the distribution of genotypes was not in HWE analyzed with Hardy-Weinberg package; NOS, the Newcastle-Ottawa Scale (NOS) values equal to the average of two NOS scores (NOS1 and NOS2) of each study assessed by two authors (Shi Z, and Tian Y).

1.4.2 The lipid profiles of individuals with different genotypes within each study

Authors	m_RR	sd_RR	n_RR	m_RK	sd_RK	n_RK	m_KK	sd_KK	n_KK
**The data of HDLC level in different genotypes' group**									
Abd,2014	0.76	0.07	49	1.05	0.21	100	1.43	0.20	86
Abellan,2010 data 1	1.20 *	0.01 *	558 *				1.12	0.04	51
Abellan,2010 data 2	1.51 *	0.02 *	575 *				1.54	0.06	52
Akao,2014 data 1	1.17	0.31	1395	1.18	0.33	1040	1.21	0.30	184
Akao,2014 data 2	1.36	0.35	1439	1.38	0.35	1127	1.40	0.34	229
Balcerzyk,2007	1.18	0.53	90				1.11 [#]	0.25 [#]	88 [#]
Cai,2017 data 1	1.11	0.25	39	1.33	0.24	62	1.29	0.26	23
Cai,2017 data 2	1.29	0.22	37	1.22	0.34	49	1.31	0.33	16
Cenarro,2003 data 1	1.17	0.32	120				1.19 [#]	0.33 [#]	90 [#]
Cenarro,2003 data 2	1.42	0.38	75				1.45 [#]	0.41 [#]	89 [#]
Chen,2009	1.34	0.38	190	1.46	0.38	215	1.47	0.52	95
Clee,2001	0.92	0.22	424	0.93	0.23	330	0.92	0.20	36
Coban,2014 data 1	1.06	0.03	105	1.13	0.03	128	1.20	0.05	34
Coban,2014 data 2	1.29	0.03	111	1.27	0.02	172	1.30	0.04	47
Delgado-Lista,2010	1.18	0.04	50				1.22 [#]	0.05 [#]	38 [#]
Deng,2008	1.35	0.35	140	1.36	0.37	228	1.42	0.29	80
Du,2020	1.59	0.62	392	1.56	0.61	466	1.47	0.57	138
Evans,2003	1.32	0.03	279	1.34	0.03	206	1.27	0.31	30
Genvigir,2008 data 1	1.43	0.38	76				1.50 [#]	0.38 [#]	148 [#]
Genvigir,2008 data 2	1.43	0.32	54				1.51 [#]	0.34 [#]	89 [#]
Ghaznavi,2018 data 1	0.99	0.21	50	1.05	0.24	40	0.97	0.17	10
Ghaznavi,2018 data 2	1.12	0.33	29	2.43	1.21	50	1.17	0.27	21
Guo,2010	1.27	0.20	57	1.28	0.17	71	1.38	0.15	16
Haghvirdizadeh,2015 data 1	1.24	0.40	94	1.27	0.54	55	1.31	0.69	15
Haghvirdizadeh,2015 data 2	1.28	0.44	56	1.19	0.46	77	1.10	0.29	32
Harada,2003	1.27	0.41	68	1.25	0.40	140	1.24	0.35	57
Hodoğlugil,2005 data 1	0.91	0.19	574	0.91	0.19	688	0.91	0.20	204
Hodoğlugil,2005 data 2	1.07	0.24	354	1.06	0.22	480	1.07	0.26	158
Huang,2007 data 1	0.98	0.25	25	0.91	0.33	26	0.83	0.21	9
Huang,2007 data 2	0.95	0.21	20	0.90	0.24	28	1.10	0.48	5
Huang,2011 data 1	1.12	0.34	66	1.23	0.29	101	1.31	0.41	39
Huang,2011 data 2	1.43	0.34	34	1.47	0.49	73	1.54	0.48	25
Katerina,2010 data 1	1.20	0.31	182	1.23	0.33	120	1.20	0.37	38
Katerina,2010 data 2	1.57	0.43	221	1.54	0.37	133	1.66	0.46	49
Katzov,2006	1.20	0.01	1427	1.20	0.01	961	1.16	0.26	156
Kitjaroentham,2007 data 1	1.28	0.23	18				1.21 [#]	0.24 [#]	22 [#]
Kitjaroentham,2007 data 2	1.34	0.32	25				1.32 [#]	0.26 [#]	47 [#]
Kitjaroentham,2007 data 3	1.15	0.22	33				1.33 [#]	0.29 [#]	63 [#]
Kitjaroentham,2007 data 4	1.4	0.44	6				1.14 [#]	0.22 [#]	15 [#]
Kolovou F,2011	1.78	0.68	157	1.81	0.66	126	1.75	0.69	25
Kolovou,2012	1.71	0.12	225	1.66	0.14	189	1.63	0.22	32
Kolovou V,2011	1.24	0.32	276	1.20	0.36	256	1.17	0.31	54
Kolovou,2016	1.71	0.75	218	1.66	0.75	185	1.58	0.45	29
Kyriakou,2007	1.26	0.30	129	1.23	0.30	104	1.25	0.34	18
Li,2005	1.12	0.33	158	1.16	0.30	174	1.15	0.38	64
Li,2009	1.06	0.15	140	1.09	0.13	170	1.12	0.17	55
Li,2012	1.04	0.21	52	1.08	0.24	74	1.15	0.17	24
Liu,2008	1.22	0.18	29	1.26	0.19	39	1.48	0.11	3
Lu,2014	1.29	0.36	343	1.24	0.34	478	1.29	0.36	178
Lu,2020 data 1	1.10	0.30	86	1.08	0.27	106	1.17	0.38	25
Lu,2020 data 2	1.04	0.28	137	1.00	0.26	239	1.00	0.23	66
Manresa,2006	1.35	0.38	657				1.36 [#]	0.40 [#]	591 [#]
Mantaring,2007	1.58	0.95	54				1.86 [#]	1.10 [#]	70 [#]
Mao,2013 data 1	0.97	0.29	88	1.05	0.26	74	1.02	0.23	37
Mao,2013 data 2	1.06	0.32	28	1.07	0.30	33	1.13	0.35	22
Mao,2013 data 3	1.05	0.64	66	0.99	0.23	75	0.97	0.25	37
Mao,2013 data 4	1.21	0.23	28	1.29	0.24	48	1.27	0.32	19

Marvaki,2014	1.71	0.75	225	1.66	0.75	191	1.98	1.71	32
Min,2014	1.33	0.49	126	1.76	0.52	139	2.06	0.52	40
Mokuno,2015 data 1	1.44	0.36	356	1.48	0.39	714	1.48	0.39	388
Mokuno,2015 data 2	1.75	0.39	869	1.78	0.38	1818	1.78	0.37	988
Porchay,2006	1.63	0.41	2603	1.62	0.43	2020	1.65	0.48	417
Porchay,2009	1.31	0.36	1597	1.32	0.35	1284	1.33	0.36	248
Saleheen,2007	1.06	0.30	80	1.10	0.28	92	1.10	0.31	28
Sandhofer,2008	1.41	0.34	350	1.38	0.33	274	1.38	0.29	64
Shi,2009 data 1	0.99	0.45	49	1.15	0.53	60	1.31	0.48	23
Shi,2009 data 2	1.41	0.51	53	1.53	0.47	66	1.56	0.53	38
Smirnov,2018	1.29	0.40	180	1.27	0.40	138	1.38	0.40	40
Sun,2005	1.14	0.23	62				1.23 #	0.25 #	186 #
Sun,2011 data 1	1.17	0.08	160	1.31	0.07	241	1.51	0.14	67
Sun,2011 data 2	1.23	0.03	182	1.38	0.02	327	1.58	0.04	131
Takagi,2002	1.14	0.03	120	1.14	0.03	229	1.14	0.03	116
Teixeira,2020	1.26	0.34	94	1.24	0.36	58	1.34	0.41	53
Wang X,2006	1.09	0.18	108	1.11	0.23	103	1.34	0.35	21
Wang Y,2006	1.12	0.33	158	1.16	0.30	174	1.15	0.38	64
Wang,2004 data 1	0.96	0.14	79	0.95	0.14	94	1.01	0.12	49
Wang,2004 data 2	1.10	0.14	76	1.15	0.19	125	1.14	0.13	77
Wang,2008	1.15	0.16	29				1.25 #	0.24 #	64 #
Wang,2009	0.96	0.26	108	1.03	0.30	122	1.07	0.25	59
Wang,2010	1.07	0.29	148	1.11	0.30	249	1.12	0.31	79
Wang,2012	2.12	0.55	63				2.38 #	0.49 #	78 #
Wang,2013	1.19	0.24	34				1.28 #	0.26 #	80 #
Wang,2016	1.08	0.17	108	1.09	0.33	122	1.17	0.21	59
Wang,2018	1.07	0.17	67	1.19	0.31	84	1.37	0.21	38
Wang,2021 data 1	1.96	0.58	43	1.84	0.42	68	1.92	0.37	34
Wang,2021 data 2	1.83	0.79	49	1.77	0.43	113	1.75	0.56	43
Woll,2005 data 1	0.90	0.01	450	0.91	0.01	327	0.99	0.03	61
Woll,2005 data 2	1.23	0.03	115	1.24	0.03	112	1.31	0.06	30
Wu,2006	0.90	0.18	28	1.14	0.30	37	1.28	0.36	22
Wu,2007 data 1	1.27	0.29	52	1.41	0.45	107	1.48	0.45	41
Wu,2007 data 2	0.87	0.19	33	0.94	0.27	57	1.07	0.30	19
Xia,2011 data 1	1.09	0.18	96	1.11	0.23	107	1.34	0.35	24
Xia,2011 data 2	1.23	0.64	51	1.20	0.68	78	1.18	0.66	33
Xiao,2004	1.31	0.36	261	1.31	0.37	344	1.46	0.33	125
Xiao,2005	1.33	0.38	385	1.35	0.39	462	1.43	0.37	172
Xiao,2012	1.27	0.28	78	1.39	0.24	107	1.76	0.41	23
Xue,2012	1.05	0.29	132	1.09	0.28	209	1.14	0.31	70
Ya,2017 data 1	1.39	0.23	48				2.06 #	0.17 #	57 #
Ya,2017 data 2	0.84	0.10	81				1.29 #	0.14 #	35 #
Ya,2017 data 3	1.33	0.15	53				1.46 #	0.20 #	47 #
Yamakawa,2004	1.40	0.25	70	1.35	0.27	160	1.47	0.29	97
Yang,2011	0.95	0.34	75	0.99	0.25	135	1.02	0.28	56
Yao,2016a data 1	1.48	0.24	98	1.45	0.25	89	1.40	0.26	20
Yao,2016a data 2	0.93	0.11	62	0.93	0.12	98	0.93	0.10	44
Yao,2016b data 1	1.06	0.31	89	1.04	0.29	128	1.01	0.24	42
Yao,2016b data 2	1.22	0.32	119	1.22	0.29	117	1.25	0.25	40
Yao,2018	1.32	0.27	146	1.34	0.34	234	1.41	0.65	74
Young-Guk,2003 data 1	1.27	0.30	108	1.28	0.32	206	1.38	0.29	89
Young-Guk,2003 data 2	0.93	0.19	43	1.02	0.23	75	1.03	0.24	32
Young-Guk,2003 data 3	0.93	0.18	14	0.99	0.21	24	1.07	0.36	10
Young-Guk,2003 data 4	1.46	0.32	154	1.44	0.33	267	1.48	0.31	112
Yu,2008	0.96	0.21	29				1.08 #	0.19 #	20 #
Yuan,2017 data 1	1.08	0.30	50	1.12	0.28	107	1.28	0.52	46
Yuan,2017 data 2	1.02	0.29	61	1.07	0.27	102	1.25	0.37	49
Zhang L,2008	0.92	0.34	83	1.01	0.31	216	0.98	0.31	112
Zhang X,2008	1.22	0.35	49				1.39 #	0.29 #	137 #
Zhao,2004a	1.32	0.33	176	1.36	0.39	234	1.44	0.32	77
Zhao,2004b data 1	1.40	0.40	170	1.50	0.40	175	1.50	0.30	76

Zhao,2004b data 2	1.30	0.40	224	1.30	0.40	295	1.40	0.40	103
Zhao,2016	1.29	0.22	13	1.26	0.28	58	1.01	1.05	55
Zhou,2013	1.35	0.36	210	1.32	0.37	300	1.41	0.31	120

The Data of LDLC level in different genotypes' group

Authors	m_RR	sd_RR	n_RR	m_RK	sd_RK	n_RK	m_KK	sd_KK	n_KK
Abd,2014	4.35	1.57	49	3.38	1.82	100	2.69	1.55	86
Abellan,2010 data 1	2.91 *	0.04 *	558 *				3.26 [#]	0.12 [#]	51 [#]
Abellan,2010 data 2	2.96 *	0.04 *	575 *				2.96 [#]	0.12 [#]	52 [#]
Akao,2014 data 1	3.59	0.72	1395	3.58	0.73	1040	3.54	0.62	184
Akao,2014 data 2	4.01	0.81	1439	3.99	0.81	1127	4.07	0.82	229
Balcerzyk,2007	3.93	1.13	90				3.73 [#]	1.23 [#]	88 [#]
Cai,2017 data 1	3.29	0.89	39	3.03	0.92	62	2.99	0.71	23
Cai,2017 data 2	2.91	0.71	37	3.01	1.01	49	3.04	0.80	16
Cenarro,2003 data 1	9.10	2.00	120				8.60 [#]	1.80 [#]	90 [#]
Cenarro,2003 data 2	9.10	2.40	75				9.10 [#]	1.90 [#]	89 [#]
Chen,2009	2.85	0.84	190	2.84	0.83	215	2.80	0.82	95
Clee,2001	4.27	0.75	424	4.35	0.83	330	4.33	0.82	36
Coban,2014 data 1	2.64	0.09	98	2.77	0.08	122	3.18	0.15	34
Coban,2014 data 2	2.95	0.08	103	2.85	0.07	159	2.85	0.13	40
Delgado-Lista,2010	2.34	0.08	50				2.30 [#]	0.09 [#]	38 [#]
Deng,2008	2.72	0.73	140	2.82	0.73	228	2.81	0.66	80
Du,2020	3.06	1.08	392	3.02	1.03	466	2.96	0.98	138
Evans,2003	4.86	0.10	279	4.86	0.13	206	5.02	0.31	30
Genvigir,2008 data 1	4.39	1.11	76				4.41 [#]	1.13 [#]	148 [#]
Genvigir,2008 data 2	2.59	0.48	54				2.55 [#]	0.44 [#]	89 [#]
Ghaznavi,2018 data 1	3.18	1.03	50	2.77	1.23	40	2.73	1.33	10
Ghaznavi,2018 data 2	2.80	0.84	29	2.52	0.88	50	2.24	0.92	21
Guo,2010	2.92	0.77	57	2.97	0.68	71	2.84	0.31	16
Haghvirdizadeh,2015 data 1	2.34	0.92	94	2.33	0.87	55	2.46	0.94	15
Haghvirdizadeh,2015 data 2	2.71	0.95	56	2.47	0.91	77	2.80	1.03	32
Harada,2003	2.94	0.93	68	2.95	1.02	140	3.08	0.71	57
Huang,2007 data 1	3.10	0.90	25	3.01	0.88	26	3.46	1.29	9
Huang,2007 data 2	3.14	0.68	20	3.30	0.90	28	3.21	1.27	5
Huang,2011 data 1	3.43	0.94	66	3.29	0.85	101	3.15	0.95	39
Huang,2011 data 2	3.37	0.99	34	3.19	1.13	73	2.99	1.15	25
Katzov,2006	4.05	0.03	1415	4.08	0.03	951	4.26	0.86	154
Kitjaroentham,2007 data 1	3.66	1.88	18				3.37 [#]	0.80 [#]	22 [#]
Kitjaroentham,2007 data 2	4.01	2.07	25				3.85 [#]	1.36 [#]	47 [#]
Kitjaroentham,2007 data 3	3.54	0.96	33				3.08 [#]	0.86 [#]	63 [#]
Kitjaroentham,2007 data 4	3.78	1.76	6				3.88 [#]	0.96 [#]	15 [#]
Kolovou F,2011	2.51	1.00	157	2.86	0.96	126	2.70	1.07	25
Kolovou,2012	2.74	0.23	225	2.69	0.26	199	3.05	0.36	32
Kolovou V,2011	5.17	1.24	276	4.81	1.12	256	5.12	0.97	54
Kolovou,2016	2.74	1.19	218	2.74	1.19	185	3	1.16	29
Li,2005	2.27	0.75	158	2.15	0.68	174	2.24	0.80	64
Li,2009	3.13	0.26	140	3.11	0.24	170	3.12	0.28	55
Li,2012	3.16	0.87	52	3.34	1.08	74	3.25	0.78	24
Liu,2008	3.07	0.56	29	2.94	0.63	39	2.81	0.57	3
Lu,2014	3.00	0.98	343	3.10	0.96	478	3.05	0.85	178
Lu,2020 data 1	2.46	2.66	86	2.44	0.76	106	2.09	0.52	25
Lu,2020 data 2	2.73	0.91	137	2.49	0.92	239	2.73	1.03	66
Manresa,2006	3.85	1.01	657				3.88 [#]	1.09 [#]	591 [#]
Mantaring,2007	3.08	1.14	54				2.97 [#]	1.32 [#]	70 [#]
Mao,2013 data 1	2.32	0.89	88	2.18	0.78	74	2.32	0.95	37
Mao,2013 data 2	2.51	0.67	28	2.42	0.84	33	2.18	0.99	22
Mao,2013 data 3	2.22	0.96	66	2.43	1.15	75	1.93	0.93	37
Mao,2013 data 4	2.31	0.72	28	2.48	0.52	48	2.59	0.54	19
Marvaki,2014	2.74	1.16	225	2.74	1.16	191	3.15	1.22	32
Min,2014	4.12	0.93	126	4.10	0.86	139	4.33	1.21	40
Sandhofer,2008	3.83	0.97	350	3.73	0.96	274	3.88	0.92	64
Shi,2009 data 1	3.46	1.01	49	3.35	0.94	60	3.05	1.10	23

Shi,2009 data 2	2.29	1.04	53	2.26	0.96	66	2.18	1.10	38
Sun,2005	2.65	0.74	62				2.92 #	0.86 #	186 #
Sun,2011 data 1	2.77	0.10	160	2.68	0.09	241	2.73	0.18	67
Sun,2011 data 2	2.89	0.08	182	2.84	0.06	327	2.89	0.09	131
Teixeira,2020	2.84	0.94	94	2.75	0.86	58	3.12	1.87	53
Wang X,2006	2.92	0.78	108	2.72	0.93	103	2.69	0.77	21
Wang Y,2006	2.27	0.75	158	2.15	0.68	174	2.24	0.80	64
Wang,2004 data 1	3.67	0.70	79	3.62	0.75	94	3.51	0.77	49
Wang,2004 data 2	3.56	0.77	76	3.54	0.95	125	3.53	0.81	77
Wang,2009	2.29	0.92	108	2.48	0.98	122	2.36	0.86	59
Wang,2010	3.03	0.87	148	3.03	0.92	249	2.98	0.77	79
Wang,2012	6.68	1.74	63				7.08 #	2.00 #	78 #
Wang,2013	2.64	0.74	34				2.97 #	0.76 #	80 #
Wang,2016	2.36	0.98	108	2.57	0.98	122	2.49	0.93	59
Wang,2018	2.36	0.97	67	2.47	0.98	84	2.49	0.93	38
Wang,2021 data 1	3.06	0.74	43	3.09	0.86	68	3.08	1.02	34
Wang,2021 data 2	2.86	0.87	49	3.26	1.00	113	3.32	1.12	43
Wu,2007 data 1	3.03	0.65	52	3.15	0.72	107	3.08	0.90	41
Wu,2007 data 2	2.66	0.85	33	2.44	0.98	57	2.24	0.79	19
Xia,2011 data 1	2.92	0.78	96	2.72	0.93	107	2.69	0.77	24
Xia,2011 data 2	3.56	0.77	51	3.54	0.95	78	3.53	0.81	33
Xiao,2004	2.82	0.83	261	2.83	0.77	344	2.95	0.78	125
Xiao,2005	2.84	0.84	385	2.83	0.79	462	2.88	0.87	172
Xiao,2012	2.35	0.72	78	2.49	0.76	107	2.44	0.75	23
Xue,2012	3.11	0.85	132	3.07	0.97	209	2.98	0.69	70
Ya,2017 data 1	2.79	0.30	48				2.63 #	0.19 #	57 #
Ya,2017 data 2	2.75	0.16	81				2.90 #	0.19 #	35 #
Ya,2017 data 3	2.95	0.21	53				2.66 #	0.22 #	47 #
Yang,2011	2.95	0.94	75	2.98	0.92	135	2.92	0.92	56
Yao,2016a data 1	2.08	0.69	98	2.18	0.81	89	2.02	0.48	20
Yao,2016a data 2	1.93	0.75	62	1.97	0.86	98	1.92	0.69	44
Yao,2016b data 1	2.48	0.63	89	2.64	0.96	128	2.52	0.71	42
Yao,2016b data 2	2.32	0.74	119	2.34	0.73	117	2.41	0.78	40
Yao,2018	2.71	0.42	146	2.76	0.56	234	2.82	0.52	74
Young-Guk,2003 data 1	3.34	0.92	108	3.17	0.92	206	3.36	0.89	89
Young-Guk,2003 data 2	3.41	1.04	43	3.21	1.13	75	3.05	1.18	32
Young-Guk,2003 data 3	3.30	0.80	14	3.40	1.57	24	3.17	0.80	10
Young-Guk,2003 data 4	3.19	0.86	154	3.39	0.88	267	3.40	1.04	112
Yu,2008	3.45	0.85	29				3.35 #	0.78 #	20 #
Yuan,2017 data 1	3.11	0.87	50	2.97	0.90	107	2.74	0.75	46
Yuan,2017 data 2	2.92	1.01	61	2.69	0.73	102	2.73	0.85	49
Zhang L,2008	2.78	0.91	83	2.96	1.18	216	2.90	0.82	112
Zhang X,2008	2.42	0.75	49				2.33 #	0.58 #	137 #
Zhao,2004a	2.93	0.80	176	2.80	0.79	234	2.80	0.68	77
Zhao,2004b data 1	2.90	0.80	170	2.80	0.80	175	3.00	0.80	76
Zhao,2004b data 2	2.80	0.80	224	2.80	0.80	295	2.80	0.90	103
Zhao,2016	2.98	0.72	13	3.03	0.52	58	2.95	0.69	55
Zhou,2013	2.82	0.81	210	2.84	0.79	300	2.93	0.72	120

The Data of TG level in different genotypes' group

Authors	m_RR	sd_RR	n_RR	m_RK	sd_RK	n_RK	m_KK	sd_KK	n_KK
Abd,2014	2.52	0.81	49	2.46	0.55	100	2.11	0.74	86
Akao,2014 data 1	1.50	0.74	1395	1.50	0.74	1040	1.44	0.59	184
Akao,2014 data 2	1.61	0.69	1439	1.55	0.64	1127	1.60	0.68	229
Balcerzyk,2007	1.80	0.98	90				1.96 #	0.99 #	88 #
Cai,2017 data 1	2.05	1.32	39	2.02	2.28	62	2.49	2.13	23
Cai,2017 data 2	2.12	1.57	37	2.03	1.04	49	2.14	1.35	16
Cenarro,2003 data 1	1.53	0.65	120				1.60 #	0.81 #	90 #
Cenarro,2003 data 2	1.46	0.64	75				1.38 #	0.63 #	89 #
Chen,2009	1.56	1.06	190	1.47	0.86	215	1.60	0.81	95
Clee,2001	1.84	0.77	424	1.78	0.78	330	1.42	0.49	36
Coban,2014 data 1	1.63	0.01	103	1.48	0.01	128	1.48	0.01	34

Coban,2014 data 2	1.40	0.01	107	1.57	0.01	171	1.66	0.01	47
Delgado-Lista,2010	0.98	0.05	50				0.82 [#]	0.06 [#]	38 [#]
Deng,2008	1.45	0.96	140	1.50	0.66	228	1.45	0.63	80
Evans,2003	3.70	0.34	279	3.25	0.36	206	2.66	0.55	30
Genvigir,2008 data 1	1.86	0.88	76				1.58 [#]	0.64 [#]	148 [#]
Genvigir,2008 data 2	0.96	0.32	54				1.86 [#]	0.88 [#]	76 [#]
Ghaznavi,2018 data 1	2.17	1.08	50	2.43	1.03	40	2.10	0.43	10
Ghaznavi,2018 data 2	1.83	0.38	29	1.83	0.48	50	1.82	1.00	21
Guo,2010	2.09	1.02	57	1.96	0.93	71	1.76	0.58	16
Haghvirdizadeh,2015 data 1	1.85	1.45	94	1.58	0.76	55	1.90	0.82	15
Haghvirdizadeh,2015 data 2	1.26	0.79	56	1.29	0.81	77	1.25	0.58	32
Harada,2003	1.20	0.57	68	1.46	0.74	140	1.40	0.87	57
Huang,2007 data 1	1.34	0.68	25	1.18	0.77	26	1.87	1.44	9
Huang,2007 data 2	1.33	0.70	20	1.38	0.44	28	0.94	0.38	5
Huang,2011 data 1	2.53	1.75	66	2.21	1.74	101	2.13	1.55	39
Huang,2011 data 2	1.75	2.64	34	1.66	1.14	73	1.50	1.02	25
Katzov,2006	1.72	0.03	1441	1.71	0.04	969	1.87	0.11	157
Kitjaroentham,2007 data 1	1.65	0.64	18				2.19 [#]	0.80 [#]	22 [#]
Kitjaroentham,2007 data 2	1.70	0.87	25				1.54 [#]	0.89 [#]	47 [#]
Kitjaroentham,2007 data 3	1.61	0.60	33				1.44 [#]	0.71 [#]	63 [#]
Kitjaroentham,2007 data 4	1.63	1.15	6				1.44 [#]	0.99 [#]	15 [#]
Kolovou F,2011	0.99	0.57	157	1.30	0.99	126	1.04	0.65	25
Kolovou,2012	1.11	0.19	225	1.11	0.2	199	1.07	0.2	32
Kolovou V,2011	1.54	0.71	276	1.64	0.81	256	2.64	1.1	54
Kolovou,2016	1.12	0.96	218	1.12	0.87	185	1.1	0.76	29
Li,2005	1.84	0.86	158	1.78	0.84	174	1.49	1.12	64
Li,2009	1.81	0.29	140	1.73	0.26	170	1.69	0.21	55
Li,2012	1.98	1.00	52	1.83	1.03	74	1.51	0.85	24
Liu,2008	2.27	1.02	29	1.98	1.04	39	1.66	0.41	3
Lu,2014	1.87	1.57	343	1.86	1.38	478	1.76	1.46	178
Lu,2020 data 1	1.45	0.73	86	1.51	0.94	106	1.04	0.49	25
Lu,2020 data 2	1.75	1.58	137	1.59	1.20	239	1.43	0.65	66
Mantaring,2007	1.82	3.32	54				1.83 [#]	3.35 [#]	70 [#]
Mao,2013 data 1	1.72	1.16	88	1.81	0.75	74	2.07	1.18	37
Mao,2013 data 2	1.65	0.74	28	2.18	1.42	33	1.85	0.86	22
Mao,2013 data 3	2.17	2.32	66	2.11	1.58	75	2.06	1.31	37
Mao,2013 data 4	1.61	0.99	28	1.89	0.77	48	1.61	0.64	19
Marvaki,2014	1.1	0.96	225	1.1	0.87	191	1.07	0.76	32
Min,2014	4.81	0.83	126	4.34	0.81	139	3.90	0.76	40
Sandhofer,2008	1.56	1.04	350	1.57	0.91	274	1.50	0.84	64
Shi,2009 data 1	1.79	1.22	49	1.70	0.91	60	1.62	1.04	23
Shi,2009 data 2	1.36	1.05	53	1.40	0.89	66	1.37	0.92	38
Smirnov,2018	4.05	8.60	180	2.90	3.30	138	2.10	1.90	40
Sun,2005	1.81	0.94	62				1.69 [#]	0.94 [#]	186 [#]
Sun,2011 data 1	2.07	0.13	160	1.66	0.11	241	1.52	0.23	67
Sun,2011 data 2	2.04	0.10	182	1.75	0.07	327	1.68	0.11	131
Takagi,2002	1.51	0.08	120	1.61	0.07	229	1.46	0.08	116
Teixeira,2020	1.47	0.77	94	1.47	0.72	58	1.36	0.82	53
Wang,2004 data 1	1.56	0.63	79	1.32	0.65	94	1.31	0.62	49
Wang,2004 data 2	1.23	0.64	76	1.20	0.68	125	1.18	0.66	77
Wang Y,2006	1.84	0.86	158	1.78	0.84	174	1.49	1.12	64
Wang,2009	1.76	1.13	108	1.91	1.61	122	1.51	1.12	59
Wang,2010	1.78	0.96	148	1.74	0.98	249	1.53	0.61	79
Wang,2012	0.27	0.03	63				0.24 [#]	0.03 [#]	78 [#]
Wang,2013	1.41	0.55	34				1.29 [#]	0.58 [#]	80 [#]
Wang,2016	1.81	1.12	108	2.01	1.72	122	1.63	1.15	59
Wang,2018	1.57	1.11	67	1.81	1.71	84	1.39	1.16	38
Wang,2021 data 1	3.31	1.37	43	3.69	1.39	68	3.43	1.40	34
Wang,2021 data 2	3.89	1.53	49	4.25	2.94	113	3.83	1.95	43
Wang X,2006	1.87	1.34	108	1.72	0.63	103	1.43	0.59	21
Wu,2007 data 2	3.82	2.02	33	3.42	1.67	57	3.33	1.43	19

Wu,2007 data 1	1.41	0.84	52	1.22	0.37	107	1.21	0.46	41
Xia,2011 data 1	1.87	1.34	96	1.72	0.63	107	1.43	0.59	24
Xia,2011 data 2	4.60	0.82	51	4.55	0.81	78	4.40	0.93	33
Xiao,2004	1.64	1.15	261	1.59	0.84	344	1.63	0.77	125
Xiao,2005	1.60	1.12	385	1.58	0.84	462	1.55	0.63	172
Xiao,2012	1.38	0.80	78	1.33	0.57	107	1.20	0.60	23
Xue,2012	2.14	0.82	132	2.00	0.92	209	1.60	0.67	70
Ya,2017 data 3	1.50	0.09	53				1.39 [#]	0.16 [#]	47 [#]
Ya,2017 data 1	1.47	0.16	48				1.15 [#]	0.10 [#]	57 [#]
Ya,2017 data 2	1.31	0.08	81				1.42 [#]	0.11 [#]	35 [#]
Yamakawa,2004	0.81	0.36	70	0.87	0.36	160	0.84	0.41	97
Yang,2011	1.69	0.62	75	1.62	0.86	135	1.65	0.73	56
Yao,2016a data 1	0.86	0.38	98	0.97	0.55	89	0.86	0.58	20
Yao,2016a data 2	1.41	0.97	62	1.2	0.8	98	1.12	0.4	44
Yao,2016b data 1	1.68	1.23	89	1.94	1.33	128	1.71	0.91	42
Yao,2016b data 2	1.16	0.9	119	1.06	0.74	117	1.01	0.62	40
Yao,2018	1.41	0.87	146	1.53	0.72	234	1.46	0.68	74
Young-Guk,2003 data 1	1.68	0.83	108	1.66	0.92	206	1.58	0.74	89
Young-Guk,2003 data 2	1.75	0.88	43	2.03	1.45	75	1.70	0.91	32
Young-Guk,2003 data 3	1.60	0.34	14	1.87	0.96	24	1.67	0.80	10
Young-Guk,2003 data 4	1.23	0.68	154	1.26	0.77	267	1.20	0.62	112
Yu,2008	1.95	0.47	29				1.63 [#]	0.38 [#]	20 [#]
Yuan,2017 data 1	2.03	1.30	50	1.68	0.85	107	1.37	0.81	46
Yuan,2017 data 2	2.03	1.30	61	1.68	0.85	102	1.37	0.81	49
Zhang L,2008	1.68	0.97	83	1.65	0.96	216	1.63	0.93	112
Zhang X,2008	1.54	1.09	49				1.30 [#]	0.45 [#]	137 [#]
Zhao,2004a	1.58	0.83	176	1.47	0.60	234	1.56	0.84	77
Zhao,2004b data 1	1.60	1.20	170	1.60	0.80	175	1.60	0.70	76
Zhao,2004b data 2	1.60	1.10	224	1.60	0.80	295	1.60	0.80	103
Zhao,2016	3.05	0.11	13	3.04	0.67	58	3.09	0.21	55
Zhou,2013	1.50	1.11	210	1.57	0.80	300	1.75	0.98	120

The Data of TC level in different genotypes' group

**Authors**	**m_RR**	**sd_RR**	**n_RR**	**m_RK**	**sd_RK**	**n_RK**	**m_KK**	**sd_KK**	**n_KK**
Abd,2014	6.26	1.14	49	5.56	1.75	100	5.09	1.70	86
Abellan,2010 data 1	5.15 *	0.05 *	558 *				5.49	0.15	51
Abellan,2010 data 2	5.21 *	0.04 *	575 *				5.21	0.14	52
Akao,2014 data 1	5.36	0.80	1395	5.36	0.80	1040	5.31	0.71	184
Akao,2014 data 2	6.00	0.89	1439	5.97	0.88	1127	6.11	0.93	229
Balcerzyk,2007	5.80	1.35	90				5.71 [#]	1.39 [#]	88 [#]
Cai,2017 data 1	5.25	1.36	39	5.05	1.71	62	5.01	1.42	23
Cai,2017 data 2	4.61	0.99	37	4.70	1.41	49	4.86	1.30	16
Cenarro,2003 data 1	10.90	2.00	120				10.50 [#]	1.70 [#]	90 [#]
Cenarro,2003 data 2	11.20	2.30	75				11.10 [#]	1.90 [#]	89 [#]
Chen,2009	4.95	0.98	190	4.96	0.98	215	5.17	0.92	95
Clee,2001	6.02	0.86	424	6.07	0.89	330	5.89	0.85	36
Coban,2014 data 1	4.65	0.11	105	4.74	0.10	128	5.19	0.13	34
Coban,2014 data 2	4.99	0.10	111	5.01	0.08	172	5.12	0.16	47
Delgado-Lista,2010	3.97	0.09	50				3.89 [#]	0.09 [#]	38 [#]
Deng,2008	4.71	0.85	140	4.79	0.92	228	4.77	0.85	80
Evans,2003	7.37	0.13	279	7.34	0.16	206	7.24	0.26	30
Genvigir,2008 data 1	6.67	1.17	76				6.64 [#]	1.23 [#]	148 [#]
Genvigir,2008 data 2	4.47	0.51	54				4.49 [#]	0.45 [#]	89 [#]
Ghaznavi,2018 data 1	5.09	1.37	50	5.11	1.49	40	6.02	2.05	10
Ghaznavi,2018 data 2	4.70	0.87	29	4.48	1.12	50	4.15	1.01	21
Guo,2010	4.88	0.80	57	4.98	0.82	71	4.92	0.64	16
Haghvirdizadeh,2015 data 1	4.42	1.19	94	4.29	1.08	55	4.45	1.11	15
Haghvirdizadeh,2015 data 2	4.66	1.16	56	4.30	1.21	77	4.34	1.04	32
Harada,2003	4.54	0.91	68	4.76	0.67	140	4.85	0.84	57
Huang,2007 data 1	4.69	0.94	25	4.55	0.97	26	5.14	1.12	9
Huang,2007 data 2	4.70	0.78	20	4.85	0.94	28	4.58	1.65	5
Huang,2011 data 1	5.53	1.00	66	5.46	0.99	101	5.37	0.91	39

Huang,2011 data 2	5.55	1.46	34	5.36	1.04	73	5.17	1.13	25
Katzov,2006	6.01	0.03	1439	6.04	0.04	969	6.24	0.09	157
Kitjaroentham,2007 data 1	5.58	1.92	18				5.60 #	0.95 #	22 #
Kitjaroentham,2007 data 2	5.95	1.90	25				5.95 #	1.47 #	47 #
Kitjaroentham,2007 data 3	5.26	0.98	33				5.23 #	0.83 #	63 #
Kitjaroentham,2007 data 4	5.68	1.95	6				5.76 #	1.13 #	15 #
Kolovou F,2011	4.85	1.53	157	5.36	1.54	126	5.01	1.49	25
Kolovou,2012	5.15	0.34	225	5.02	0.36	199	5.3	0.54	32
Kolovou V,2011	7.54	1.55	276	7.15	1.29	256	7.2	0.9	54
Kolovou,2016	5.2	1.68	218	5.28	1.76	185	5.35	1.68	29
Li,2005	4.47	1.11	158	4.40	1.13	174	4.39	0.99	64
Li,2009	5.12	0.51	140	5.11	0.48	170	5.13	0.52	55
Li,2012	5.00	0.88	52	5.21	1.27	74	5.29	0.97	24
Liu,2008	4.97	0.85	29	4.87	0.86	39	4.93	0.66	3
Lu,2014	4.81	1.03	343	4.89	1.19	478	4.86	0.85	178
Lu,2020 data 1	4.15	0.96	86	4.08	1.01	106	3.65	0.72	25
Lu,2020 data 2	4.39	1.18	137	4.06	1.14	239	4.25	1.30	66
Manresa,2006	5.77	1.14	657				5.74 #	1.16 #	591 #
Mantaring,2007	5.30	1.33	54				5.48 #	1.54 #	70 #
Mao,2013 data 1	4.15	1.09	88	4.1	0.84	74	4.36	1.08	37
Mao,2013 data 2	4.06	0.77	28	4.46	1	33	3.89	0.87	22
Mao,2013 data 3	4.23	1.44	66	4.33	1.26	75	3.97	0.83	37
Mao,2013 data 4	3.92	0.77	28	4.1	0.57	48	4.02	0.63	19
Marvaki,2014	5.17	1.66	225	5.25	1.73	191	5.51	1.76	32
Min,2014	6.12	1.31	126	5.76	1.59	139	5.99	1.48	40
Sandhofer,2008	5.96	1.08	350	5.83	1.04	274	5.91	0.93	64
Shi,2009 data 1	5.46	0.95	49	5.39	1.12	60	5.36	1.22	23
Shi,2009 data 2	4.50	0.94	53	4.36	1.02	66	4.30	1.14	38
Smirnov,2018	7.89	3.10	180	7.56	3.80	138	7.97	2.10	40
Sun,2005	4.64	1.08	62				4.94 #	1.03 #	186 #
Sun,2011 data 1	4.66	0.15	160	4.54	0.13	241	5.00	0.27	67
Sun,2011 data 2	4.44	0.08	182	4.50	0.06	327	4.51	0.10	131
Takagi,2002	5.33	0.1	120	5.4	0.07	229	5.28	0.09	116
Teixeira,2020	4.84	1.32	94	4.65	1.02	58	4.76	1.16	53
Wang,2004 data 1	4.57	0.82	79	4.57	0.88	94	4.56	0.79	49
Wang,2004 data 2	4.60	0.82	76	4.55	0.81	125	4.40	0.93	77
Wang X,2006	4.58	0.83	108	4.43	0.93	103	4.37	1.22	21
Wang Y,2006	4.47	1.11	158	4.40	1.13	174	4.39	0.99	64
Wang,2009	4.93	1.29	108	5.05	1.34	122	4.40	1.13	59
Wang,2010	4.79	0.95	148	4.85	1.07	249	4.88	0.93	79
Wang,2012	9.95	1.95	63				10.57 #	2.41 #	78 #
Wang,2013	4.65	1.08	34				4.94 #	1.03 #	80 #
Wang,2016	5.01	1.31	108	5.38	1.41	122	4.67	1.11	59
Wang,2018	4.55	1.29	67	4.36	1.41	84	4.08	1.11	38
Wang,2021 data 1	6.49	1.14	43	6.41	1.12	68	6.33	1.50	34
Wang,2021 data 2	5.85	1.46	49	6.33	1.45	113	6.48	1.78	43
Wu,2006	4.84	1.01	28	4.79	0.99	37	3.89	1.17	22
Wu,2007 data 1	5.03	0.72	52	5.16	0.71	107	5.20	0.86	41
Wu,2007 data 2	5.25	0.66	33	4.89	1.09	57	4.89	0.73	19
Xia,2011 data 1	4.58	0.83	96	4.43	0.93	107	4.37	1.22	24
Xia,2011 data 2	4.60	0.82	51	4.55	0.81	78	4.40	0.93	33
Xiao,2004	4.79	0.98	261	4.75	0.99	344	5.02	0.97	125
Xiao,2005	4.81	1.05	385	4.78	0.99	462	4.87	1.03	172
Xiao,2012	4.22	0.97	78	4.34	0.99	107	4.31	0.96	23
Xue,2012	4.94	0.99	132	5.04	1.12	209	5.01	0.95	70
Ya,2017 data 1	4.86	0.23	48				4.21 #	0.12 #	57 #
Ya,2017 data 2	4.26	0.07	81				4.60 #	0.12 #	35 #
Ya,2017 data 3	4.43	0.10	53				4.89 #	0.08 #	47 #
Yang,2011	4.92	1.06	75	4.65	0.99	135	4.61	0.91	56
Yao,2016a data 1	4.16	0.92	98	4.23	0.9	89	4.18	0.86	20
Yao,2016a data 2	3.75	1.29	62	3.62	1.08	98	3.63	0.87	44

Yao,2016b data 1	4.5	0.9	89	4.82	1.3	128	4.75	0.65	42
Yao,2016b data 2	4.32	0.97	119	4.23	0.75	117	4.31	0.98	40
Yao,2018	4.72	0.76	146	4.82	0.88	234	1.46	0.68	74
Young-Guk,2003 data 1	5.38	1.04	108	5.22	0.97	206	5.44	0.96	89
Young-Guk,2003 data 2	5.40	0.80	43	5.32	1.06	75	5.17	0.93	32
Young-Guk,2003 data 3	5.45	0.72	14	5.70	0.59	24	4.94	0.44	10
Young-Guk,2003 data 4	5.21	0.94	154	5.41	1.01	267	5.42	1.11	112
Yu,2008	5.63	1.31	29				5.35 #	1.29 #	20 #
Yuan,2017 data 1	4.67	1.18	50	4.50	1.20	107	4.32	1.19	46
Yuan,2017 data 2	4.61	1.45	61	4.08	0.89	102	4.11	1.01	49
Zhang L,2008	4.65	0.96	83	4.74	1.02	216	4.62	1.06	112
Zhang X,2008	4.67	0.84	49				4.69 #	0.98 #	137 #
Zhao,2004a	4.92	1.01	176	4.79	1.01	234	4.82	0.99	77
Zhao,2004b data 1	4.90	1.00	170	4.90	1.00	175	5.10	1.00	76
Zhao,2004b data 2	4.80	1.10	224	4.70	1.00	295	4.70	1.00	103
Zhao,2016	4.88	1.01	13	4.81	0.91	58	4.72	1.03	55
Zhou,2013	4.78	0.97	210	4.76	0.99	300	4.99	0.91	120

Supplementary Table S2. The lipid levels of different genotypes' group in each study. **Abbreviation:** m_, the mean of lipid level; sd_, the standard deviation of lipid level; n_, the number of individuals; * the level of combined group of RR and RK genotypes individuals; # the level of combined group of KK and RK genotypes individuals.

2 RESULTS

2.1 Effect of R219K on HDLC level

2.1.1 Influence analysis

It indicated that six samples ([23]Abd 2014, males in [1]Coban 2014, two sample of [24] Sun,2011, and AD and PD patients in Ya, 2017[25]) may distort the pooled effect of R219K because of their extreme effect (outliers, Figure S1).

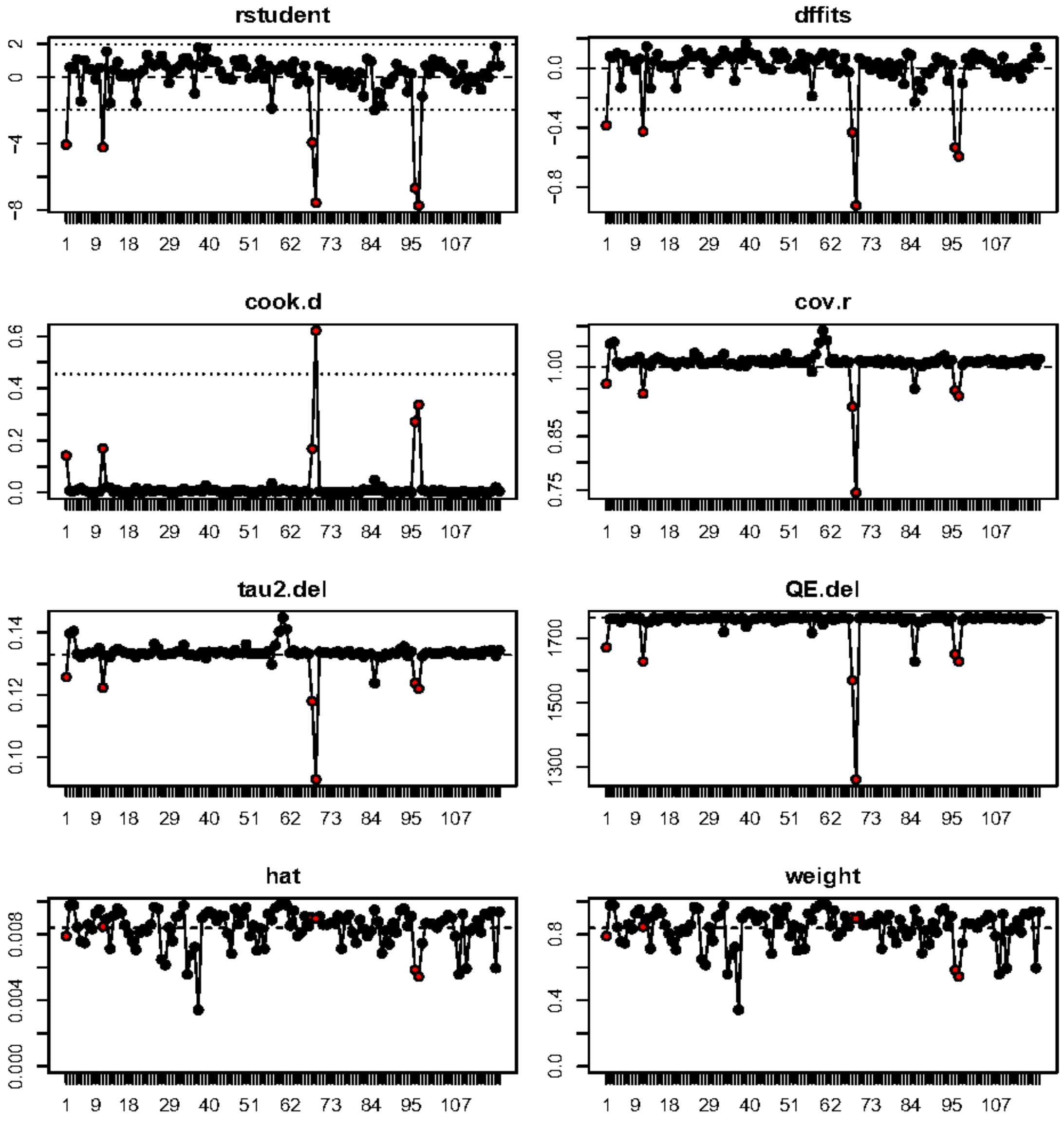

Supplementary Figure S1. Plot of the various diagnostic measures of influence analyses for relationship between R219K and HDLC level. The influence measures of each study: **retudent**, the externally standardized residuals; **diffits**, DFFITS values; **cook.d**, Cook's distances; **cov.r**, covariance ratios; **tau2.del**, estimates of T^2; **QE.del**, the test statistics for (residual) heterogeneity when each study is removed in turn; **hat**, the diagonal elements of the hat matrix; **weight**, the weights (in%) given to the observed outcomes during the model fitting. Study considered to be influential, was colored in red in the plot.

2.1.2 Subgroup analysis

Genetic models	Variables	Estimated pooled effect size				Heterogeneity		
		N	SMD	95% CI	P	Q	I^2	P
Codominant 1 (RK vs. KK)								
	Mix	20	-0.44	-0.67 ~ -0.22	**< 0.01**	272	0.93	**< 0.01**
	Patients	41	-0.10	-0.18 ~ -0.02	**0.02**	91	0.56	**< 0.01**
	Random	34	-0.10	-0.18 ~ -0.01	**0.02**	115	0.71	**< 0.01**
	Between groups:	Q = 8.39, df = 2, P = **0.02**						
	Asian	69	-0.16	-0.22 ~ -0.10	**< 0.01**	158	0.57	**< 0.01**
	Caucasian	26	-0.20	-0.38 ~ -0.01	**< 0.01**	351	0.93	**< 0.01**
	Between groups:	Q = 0.15, df = 1, P = 0.70						
Codominant 2 (RR vs. KK)								
	Mix	18	-0.35	-0.52 ~ -0.18	**< 0.01**	123	0.86	**< 0.01**
	Patients	41	-0.21	-0.32 ~ -0.09	**< 0.01**	166	0.76	**< 0.01**
	Random	34	-0.17	-0.27 ~ -0.07	**< 0.01**	161	0.80	**< 0.01**
	Between groups:	Q = 3.06, df = 2, P = 0.22						
	Asian	69	-0.29	-0.37 ~ -0.21	**< 0.01**	272	0.75	**< 0.01**
	Caucasian	24	-0.05	-0.18 ~ 0.08	0.46	146	0.84	**< 0.01**
	Between groups:	Q = 9.71, df = 1, P = < **0.01**						
Codominant 3 (RK vs. RR)								
	Mix	18	0.08	-0.02 ~ 0.19	0.11	81	0.79	**< 0.01**
	Patients	42	0.14	0.04 ~ 0.24	**< 0.01**	302	0.86	**< 0.01**
	Random	34	0.03	-0.02 ~ 0.08	0.28	91	0.64	**< 0.01**
	Between groups:	Q = 3.61, df = 2, P = 0.16						
	Asian	69	0.10	0.06 ~ 0.15	**< 0.01**	155	0.56	**< 0.01**
	Caucasian	25	0.06	-0.04 ~ 0.17	0.22	336	0.93	**< 0.01**
	Between groups:	Q = 0.40, df = 1, P = 0.53						
Dominant model (RR + RK vs. KK)								
	Mix	20	-0.49	-0.74 ~ -0.24	**< 0.01**	378	0.95	**< 0.01**
	Patients	41	-0.15	-0.24 ~ -0.05	**< 0.01**	139	0.71	**< 0.01**
	Random	35	-0.16	-0.27 ~ -0.07	**< 0.01**	223	0.85	**< 0.01**
	Between groups:	Q = 6.81, df = 2, P = **0.03**						
	Asian	69	-0.21	-0.28 ~ -0.15	**< 0.01**	226	0.70	**< 0.01**
	Caucasian	27	-0.24	-0.45 ~ -0.04	**0.02**	539	0.95	**< 0.01**
	Between groups:	Q = 0.07, df = 1, P = 0.79						
Recessive model (RR vs. RK + KK)								
	Mix	20	-0.14	-0.24 ~ -0.05	**< 0.01**	88	0.78	**< 0.01**
	Patients	50	-0.15	-0.23 ~ -0.06	**< 0.01**	302	0.84	**< 0.01**
	Random	43	-0.11	-0.17 ~ -0.05	**< 0.01**	167	0.75	**< 0.01**
	Between groups:	Q = 0.71, df = 2, P = 0.70						
	Asian	80	-0.17	-0.22 ~ -0.12	**< 0.01**	253	0.69	**< 0.01**
	Caucasian	33	-0.08	-0.16 ~ 0.00	0.05	288	0.89	**< 0.01**
	Between groups:	Q = 3.58, df = 1, P = 0.06						
Over dominant model (RK vs. RR+ KK)								
	Mix	18	-0.02	-0.08 ~ 0.03	0.41	31	0.45	**0.02**
	Patients	42	0.03	-0.02 ~ 0.07	0.23	70	0.41	**< 0.01**
	Random	35	-0.02	-0.05 ~ 0.02	0.41	52	0.34	**0.026**
	Between groups:	Q = 2.70, df = 2, P = 0.26						
	Asian	71	0.01	-0.02 ~ 0.04	0.53	106	0.34	**< 0.01**
	Caucasian	24	-0.02	-0.07 ~ 0.02	0.26	48	0.52	**< 0.01**
	Between groups:	Q = 1.63, df = 1, P = 0.20						

Supplementary Table S3. Subgroup meta-analysis for relationship between R219K polymorphism and HDLC. level Bold indicates statistically significant (p < 0.05); SMD, standard mean difference; CI confidence interval.

2.1.3 Forest of pooled effect of R219K on HDLC (in recessive model)

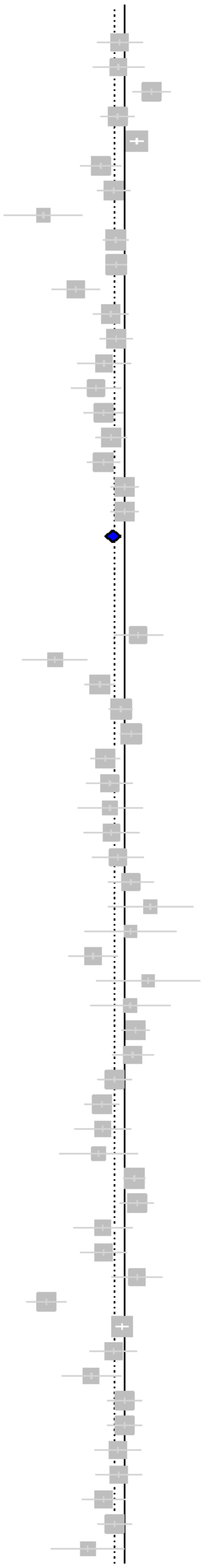

Subgroup	Standardised Mean Difference	SMD	95%−CI
**mix**			
Cenarro,2003 data 1		*−0.06*	*[−0.33; 0.21]*
Cenarro,2003 data 2		*−0.08*	*[−0.38; 0.23]*
Coban,2014 data 2		*0.33*	*[0.10; 0.56]*
Deng,2008		*−0.09*	*[−0.29; 0.11]*
Katzov,2006		*0.15*	*[0.07; 0.23]*
Wang,2009		*−0.29*	*[−0.53; −0.05]*
Wang,2010		*−0.13*	*[−0.33; 0.06]*
Wu,2006		*−1.00*	*[−1.48; −0.53]*
Xiao,2004		*−0.11*	*[−0.26; 0.04]*
Xiao,2005		*−0.10*	*[−0.23; 0.02]*
Xiao,2012		*−0.60*	*[−0.89; −0.31]*
Xue,2012		*−0.17*	*[−0.38; 0.04]*
Yao,2018		*−0.10*	*[−0.30; 0.09]*
Yuan,2017 data 1		*−0.25*	*[−0.57; 0.07]*
Yuan,2017 data 2		*−0.35*	*[−0.65; −0.05]*
Zhang,2008		*−0.26*	*[−0.50; −0.02]*
Zhao,2004a		*−0.17*	*[−0.35; 0.02]*
Zhao,2004b data 1		*−0.26*	*[−0.46; −0.07]*
Zhao,2004b data 2		*0.00*	*[−0.16; 0.16]*
Zhou,2013		*0.00*	*[−0.17; 0.17]*
**Random effects model**		**−0.14**	**[−0.24; −0.05]**
I^2 = 78% [67%; 86%], χ^2_{19} = 87.8 (p < 0.01)			
**patient**			
Balcerzyk,2007		*0.17*	*[−0.13; 0.46]*
Cai,2017 data 1		*−0.86*	*[−1.25; −0.46]*
Chen,2009		*−0.30*	*[−0.49; −0.12]*
Clee,2001		*−0.04*	*[−0.18; 0.10]*
Du,2020		*0.08*	*[−0.04; 0.21]*
Evans,2003		*−0.24*	*[−0.41; −0.06]*
Genvigir,2008 data 1		*−0.18*	*[−0.46; 0.09]*
Ghaznavi,2018 data 1		*−0.18*	*[−0.57; 0.21]*
Guo,2010		*−0.16*	*[−0.50; 0.17]*
Haghvirdizadeh,2015 data 1		*−0.08*	*[−0.39; 0.23]*
Harada,2003		*0.08*	*[−0.20; 0.35]*
Huang,2007 data 1		*0.32*	*[−0.20; 0.83]*
Huang,2007 data 2		*0.07*	*[−0.48; 0.63]*
Huang,2011 data 1		*−0.39*	*[−0.68; −0.09]*
Kiijaroentham,2007 data 1		*0.29*	*[−0.34; 0.92]*
Kiijaroentham,2007 data 2		*0.07*	*[−0.42; 0.56]*
Kolovou,2013		*0.13*	*[−0.03; 0.30]*
Kyriakou,2007		*0.10*	*[−0.15; 0.35]*
Li,2005		*−0.12*	*[−0.32; 0.08]*
Li,2009		*−0.28*	*[−0.49; −0.07]*
Li,2012		*−0.27*	*[−0.60; 0.07]*
Liu,2008		*−0.32*	*[−0.80; 0.16]*
Lu,2014		*0.12*	*[−0.01; 0.25]*
Lu,2020 data 2		*0.15*	*[−0.05; 0.36]*
Mantaring,2007		*−0.27*	*[−0.62; 0.09]*
Mao,2013 data 1		*−0.26*	*[−0.54; 0.02]*
Mao,2013 data 3		*0.15*	*[−0.15; 0.46]*
Min,2014		*−0.96*	*[−1.20; −0.72]*
Porchay,2009		*−0.03*	*[−0.10; 0.04]*
Saleheen,2007		*−0.14*	*[−0.42; 0.15]*
Shi,2009 data 1		*−0.41*	*[−0.77; −0.05]*
Smirnov,2018		*0.00*	*[−0.21; 0.21]*
Takagi,2002		*0.00*	*[−0.21; 0.21]*
Teixeira,2020		*−0.08*	*[−0.36; 0.19]*
Wang,2004 data 1		*−0.07*	*[−0.35; 0.20]*
Wang,2006		*−0.26*	*[−0.52; 0.00]*
Wang,2006		*−0.12*	*[−0.32; 0.08]*
Wang,2008		*−0.45*	*[−0.90; −0.01]*

(*Continued*)

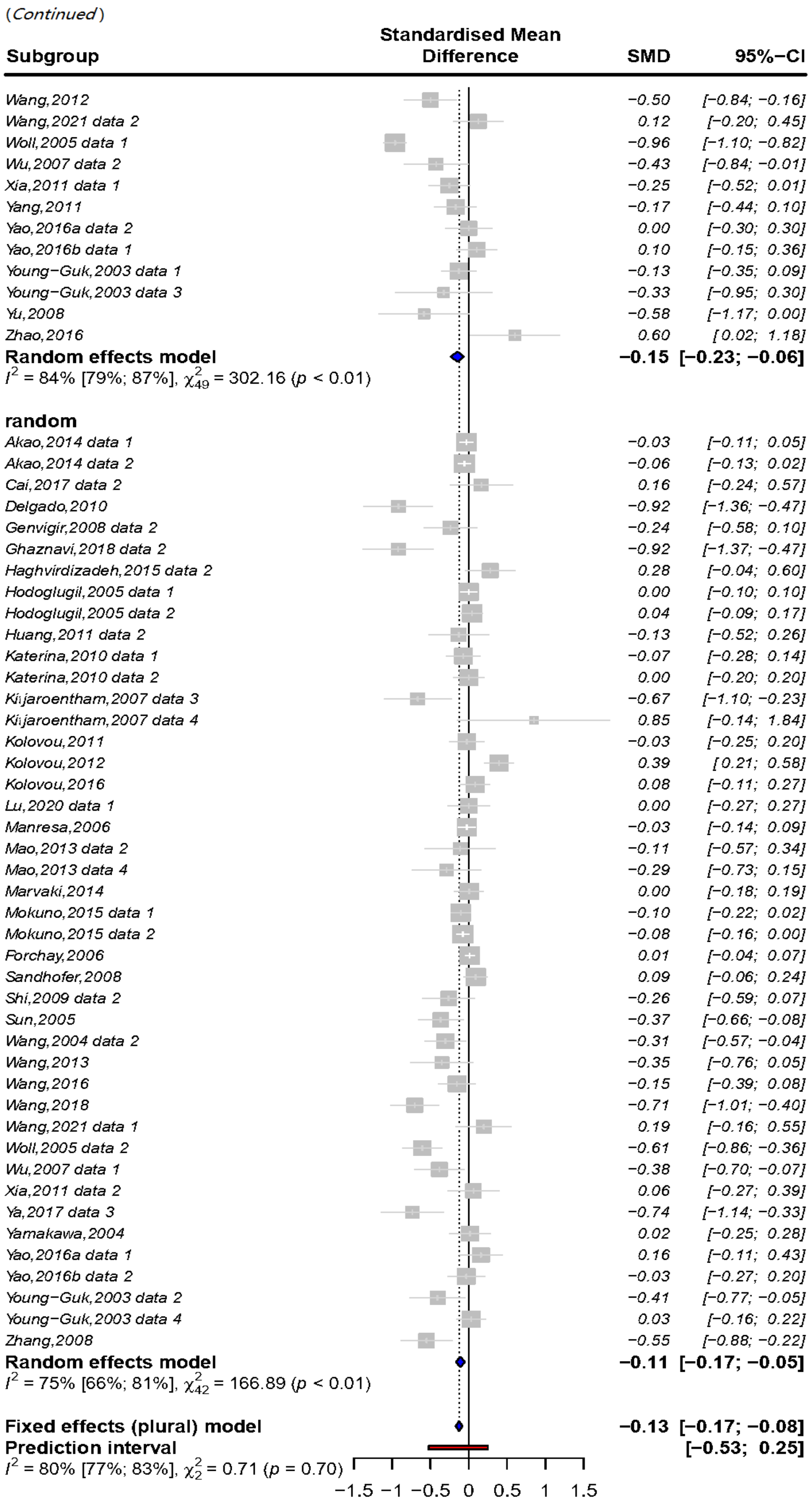

Supplementary Figure S2. The effects of R219K on HDLC level under recessive genetic model (patients: SMD = -0.15, 95%CI: -0.23 ~ -0.06; z = -3.46, P < 0.01; random: SMD = -0.11, 95%CI: -0.17 ~ -0.05; z = -3.60, P < 0.01; mix: SMD = -0.14, 95%CI: -0.24 ~ -0.05; z = -2.98, P < 0.01; and overall: SMD = -0.13, 95%CI: -0.17 ~ -0.08; z = -6.11, P < 0.01.)

2.1.4 Funnel plot of publication bias

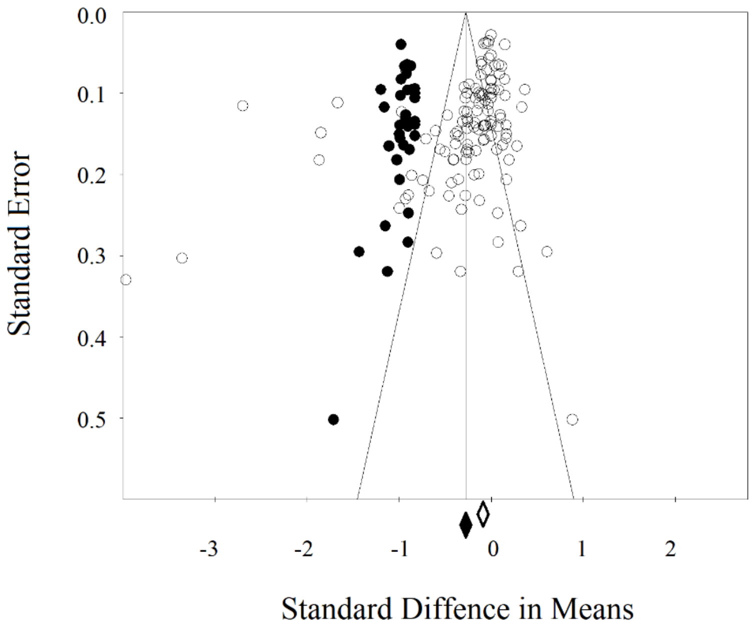

Supplementary Figure S3. Figure S3 showed the asymmetry of funnel plot for the publications of eligible studies (the imputed studies, that were not published, were indicated with solid circle; the adjusted effect size was indexed with solid diamond)

2.2 Effect of R219K on LDLC level

2.2.1 Influence analysis

It indicated that three samples (males and females in [1]Coban 2014, [24]Sun 2011 and random sample in [25]Ya 2017) may distort the pooled effect of R219K because of their extreme effect (outliers, Figure S4)

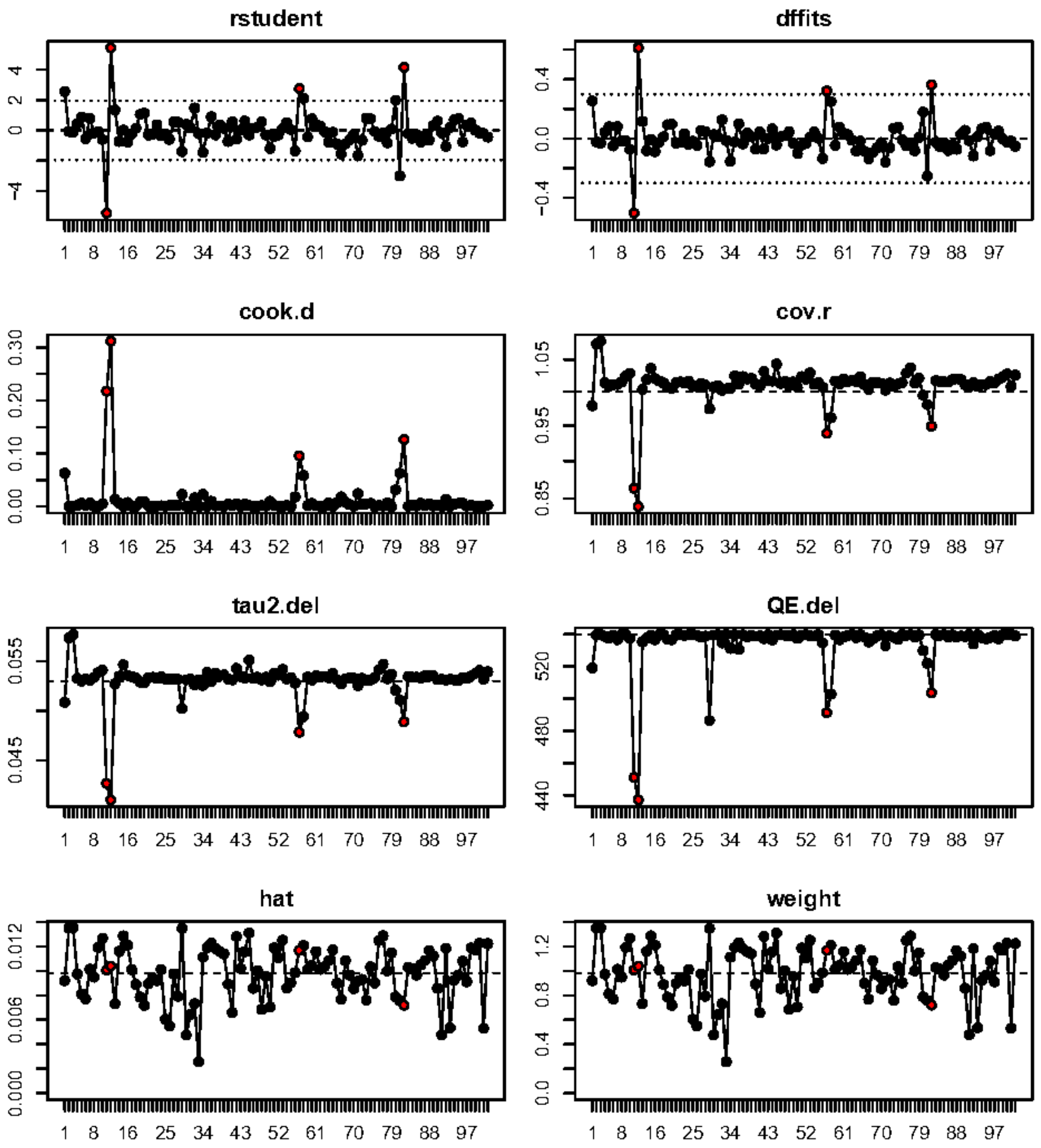

Supplementary Figure S4. Plot of the various diagnostic measures of influence analyses for relationship between R219K and LDLC level. The influence measures of each study: **retudent**, the externally standardized residuals; **diffits**, DFFITS values; **cook.d**, Cook's distances; **cov.r**, covariance ratios; **tau2.del**, estimates of T^2; **QE.del**, the test statistics for (residual) heterogeneity when each study is removed in turn; **hat**, the diagonal elements of the hat matrix; **weight**, the weights (in%) given to the observed outcomes during the model fitting. Study considered to be influential, was colored in red in the plot.

2.3 Effect of R219K on TC level

2.3.1 Influence analysis

It indicated that five samples (males in [1]Coban 2014, [26]Katzov,2006, AD and PD patients and control samples in[25] Ya, 2017) may distort the pooled effect of R219K because of their extreme effect (outliers, Figure S5).

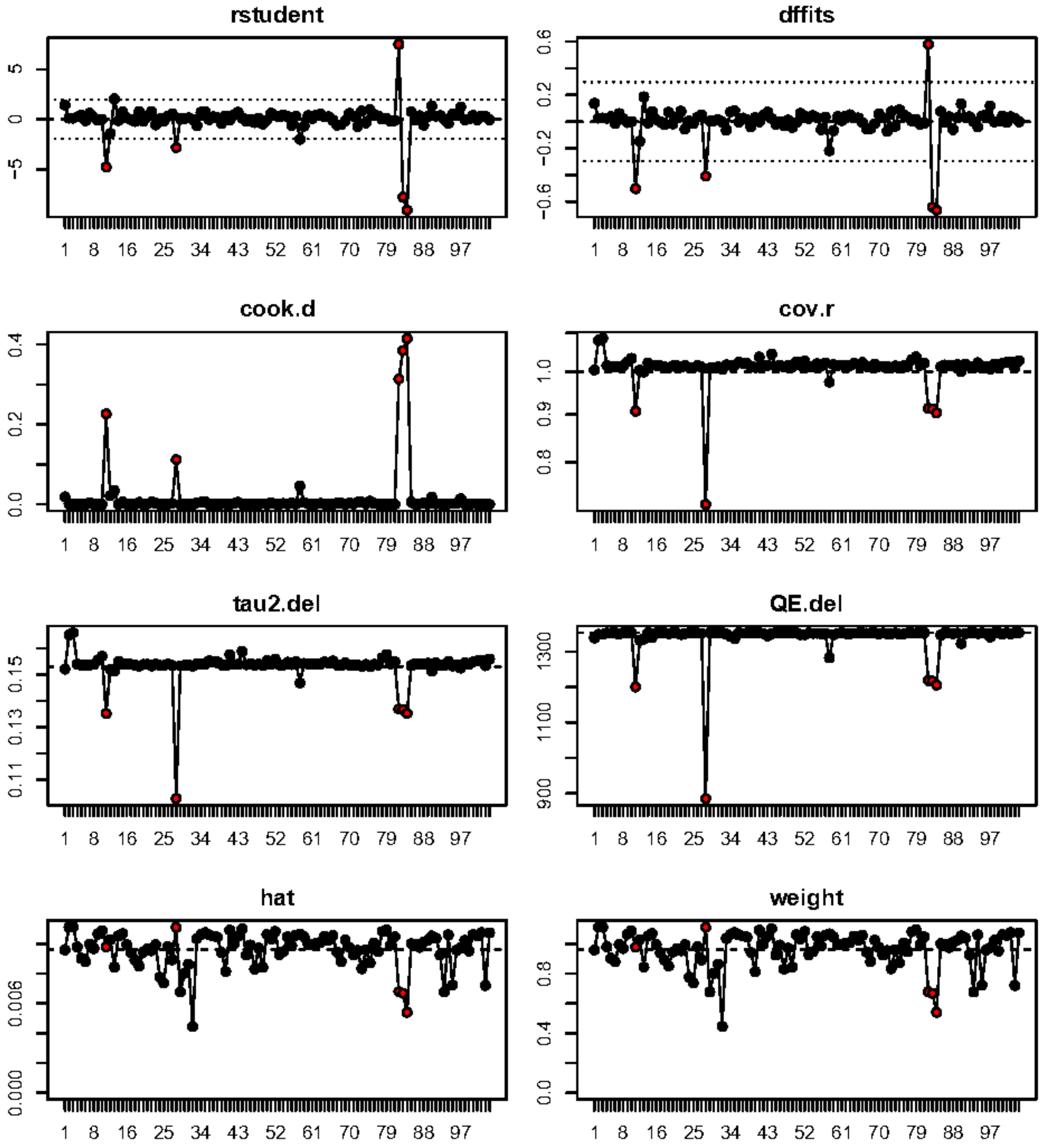

Supplementary Figure S5. Plot of the various diagnostic measures of influence analyses for relationship between R219K and TC level. The influence measures of each study: **retudent**, the externally standardized residuals; **diffits**, DFFITS values; **cook.d**, Cook's distances; **cov.r**, covariance ratios; **tau2.del**, estimates of T^2; **QE.del**, the test statistics for (residual) heterogeneity when each study is removed in turn; **hat**, the diagonal elements of the hat matrix; **weight**, the weights (in%) given to the observed outcomes during the model fitting. Study considered to be influential, was colored in red in the plot.

2.4 Effect of R219K on TG level

2.4.1 Influence analysis

It indicated that six samples (males and females in [1]Coban 2014, [27]Delgado 2010; two sample of [24]Sun,2011, and AD patients in [25]Ya, 2017) may distort the pooled effect of R219K because of their extreme effect (outliers, Figure S6).

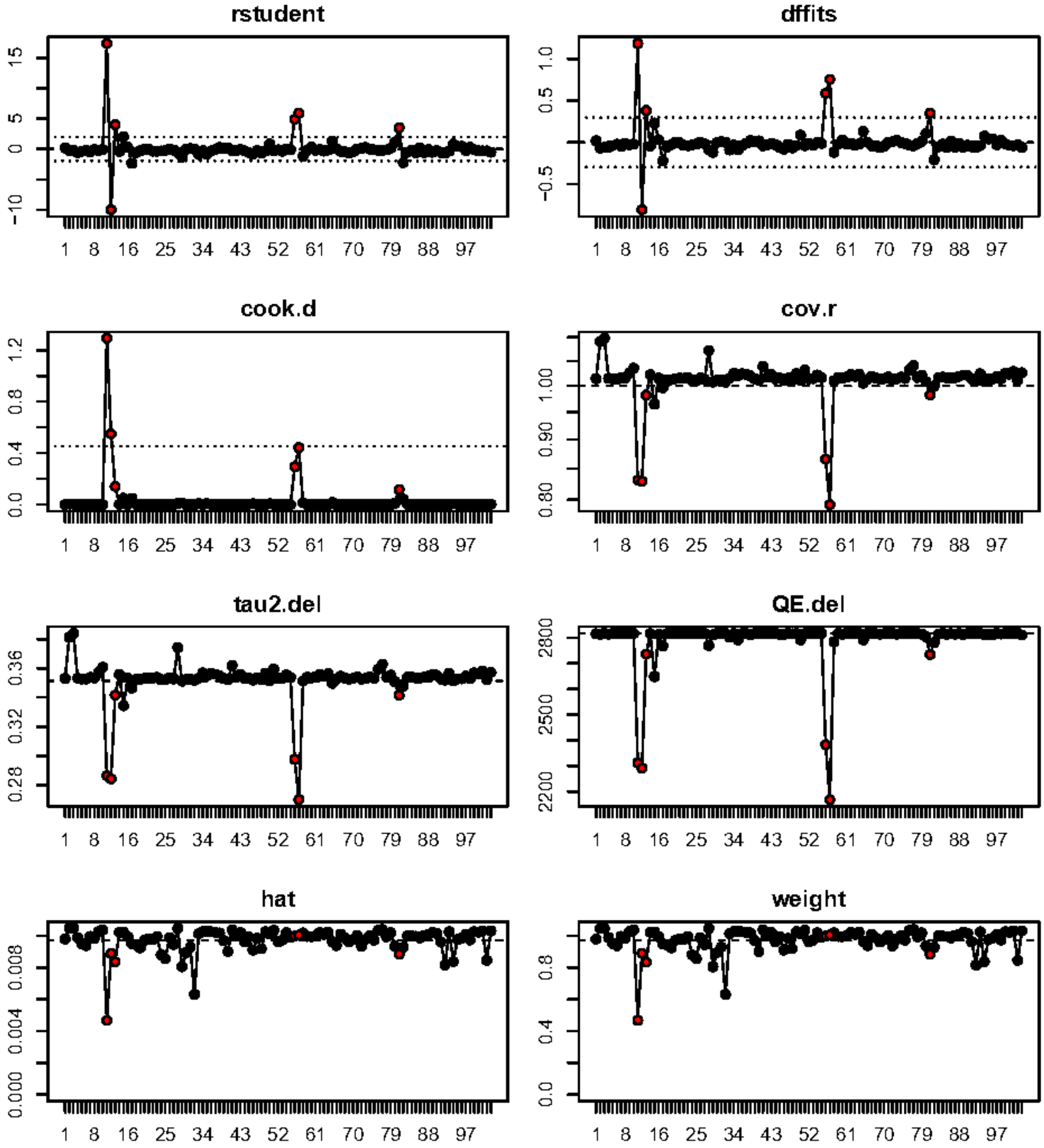

Supplementary Figure S6. Plot of the various diagnostic measures of influence analyses for relationship between R219K and TG level. The influence measures of each study: **retudent**, the externally standardized residuals; **diffits**, DFFITS values; **cook.d**, Cook's distances; **cov.r**, covariance ratios; **tau2.del**, estimates of T^2; **QE.del**, the test statistics for (residual) heterogeneity when each study is removed in turn; **hat**, the diagonal elements of the hat matrix; **weight**, the weights (in%) given to the observed outcomes during the model fitting. Study considered to be influential, was colored in red in the plot.

2.4.2 Subgroup analysis

Genetic models	Variables	Estimated pooled effect size				Heterogeneity		
		N	SMD	95% CI	P	Q	I^2	P
Codominant 1 (RK vs. KK)								
	Mix	17	0.12	0.02 ~ 0.22	**0.02**	38.3	0.58	< **0.01**
	Patients	37	0.19	0.01 ~ 0.38	**0.04**	341.5	0.89	< **0.01**
	Random	28	0.20	0.08 ~ -0.31	< **0.01**	93.0	0.71	< **0.01**
	Between groups:			Q = 1.22, df = 2, P = 0.54				
	Asian	66	0.18	0.09 ~ 0.28	< **0.01**	342	0.81	< **0.01**
	Caucasian	16	0.16	-0.07 ~ 0.39	0.17	132	0.89	< **0.01**
	Between groups:			Q = 0.03, df = 1, P = 0.86				
Codominant 2 (RR vs. KK)								
	Mix	16	0.16	0.03 ~ 0.29	**0.01**	48.6	0.69	< **0.01**
	Patients	37	0.22	0.03 ~ 0.41	**0.02**	320.4	0.89	< **0.01**
	Random	26	0.06	0.00 ~ 0.13	**0.04**	12.5	0.00	0.98
	Between groups:			Q = 3.66, df = 2, P = 0.16				
	Asian	64	0.15	0.08 ~ 0.22	< **0.01**	135	0.53	< **0.01**
	Caucasian	15	0.22	-0.12 ~ 0.55	0.20	255	0.95	< **0.01**
	Between groups:			Q = 0.14, df = 1, P = 0.71				
Codominant 3 (RK vs. RR)								
	Mix	17	-0.06	-0.14 ~ 0.02	0.12	44.2	0.64	< **0.01**
	Patients	37	-0.07	-0.20 ~ 0.07	0.32	333.0	0.89	< **0.01**
	Random	26	0.03	-0.02 ~ 0.09	0.22	35.0	0.29	0.09
	Between groups:			Q = 4.86, df = 2, P = 0.09				
	Asian	64	-0.01	-0.08 ~ 0.05	0.72	214	0.71	< **0.01**
	Caucasian	16	-0.09	-0.23 ~ 0.06	0.23	198	0.92	< **0.01**
	Between groups:			Q = 0.89, df = 1, P = 0.34				
Dominant model (RR + RK vs. KK)								
	Mix	18	0.08	-0.14 ~ 0.29	0.50	226	0.92	< **0.01**
	Patients	37	0.20	0.02 ~ 0.37	**0.03**	351	0.90	< **0.01**
	Random	28	0.20	0.05 ~ 0.34	**0.01**	172	0.84	< **0.01**
	Between groups:			Q = 0.97, df = 2, P = 0.62				
	Asian	66	0.19	0.10 ~ 0.28	< **0.01**	365	0.82	< **0.01**
	Caucasian	17	0.09	-0.25 ~ 0.44	0.59	385	0.96	< **0.01**
	Between groups:			Q = 0.27, df = 1, P = 0.60				
Recessive model (RR vs. RK + KK)								
	Mix	19	0.05	-0.03 ~ 0.13	0.20	54.4	0.67	< **0.01**
	Patients	45	0.11	-0.01 ~ 0.22	0.07	357.1	0.88	< **0.01**
	Random	33	-0.01	-0.08 ~ 0.07	0.86	95.0	0.66	< **0.01**
	Between groups:			Q = 2.87, df = 2, P = 0.24				
	Asian	75	0.08	0.02 ~ 0.13	< **0.01**	228	0.68	< **0.01**
	Caucasian	22	0.02	-0.12 ~ 0.16	0.78	301	0.93	< **0.01**
	Between groups:			Q = 0.57, df = 2, P = 0.45				
Over dominant model (RK vs. RR+ KK)								
	Mix	17	-0.00	-0.10 ~ 0.09	0.94	80.6	0.80	< **0.01**
	Patients	36	-0.06	-0.14 ~ 0.01	0.07	96.2	0.64	< **0.01**
	Random	27	0.04	-0.06 ~ 0.13	0.47	155.3	0.83	< **0.01**
	Between groups:			Q = 2.98, df = 2, P = 0.22				
	Asian	64	-0.01	-0.06 ~ 0.04	0.76	178	0.65	< **0.01**
	Caucasian	16	-0.05	-0.17 ~ 0.07	0.42	145	0.90	< **0.01**
	Between groups:			Q = 0.37, df = 1, P = 0.54				

Supplementary Table S4. Meta-analysis of the association of ABCA1 R219K polymorphism and TG level.
Bold indicates statistically significant (P < 0.05); SMD, standard mean difference; CI confidence interval.

2.4.3 Forest of pooled effect of R219K on TG (in dominant model)

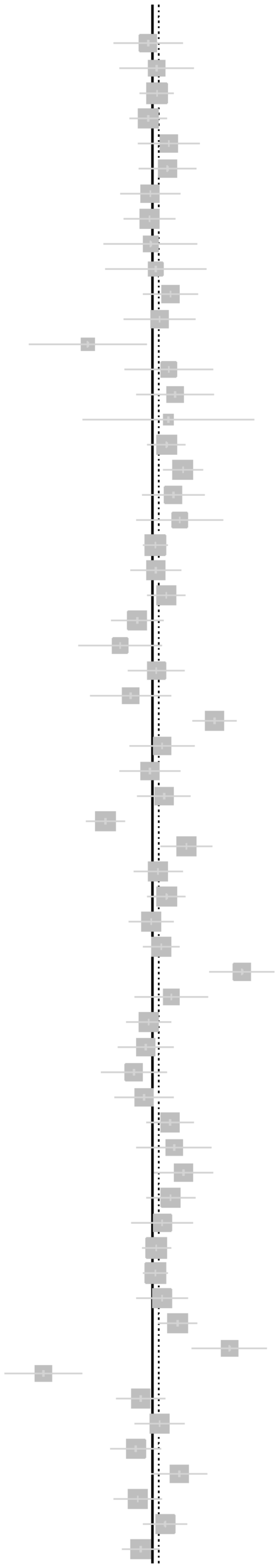

Subgroup	SMD	95%−CI
**Asian**		
Cai,2017 data 1	−0.05	[−0.43; 0.33]
Cai,2017 data 2	0.05	[−0.36; 0.45]
Chen,2009	0.05	[−0.13; 0.23]
Deng,2008	−0.05	[−0.25; 0.15]
Guo,2010	0.18	[−0.15; 0.51]
Haghvirdizadeh,2015 data 1	0.16	[−0.15; 0.47]
Haghvirdizadeh,2015 data 2	−0.03	[−0.35; 0.30]
Harada,2003	−0.03	[−0.31; 0.24]
Huang,2007 data 1	−0.02	[−0.54; 0.49]
Huang,2007 data 2	0.04	[−0.52; 0.59]
Huang,2011 data 1	0.20	[−0.09; 0.49]
Huang,2011 data 2	0.08	[−0.31; 0.47]
Kiijaroentham,2007 data 1	−0.72	[−1.37; −0.08]
Kiijaroentham,2007 data 2	0.18	[−0.31; 0.67]
Kiijaroentham,2007 data 3	0.25	[−0.17; 0.67]
Kiijaroentham,2007 data 4	0.18	[−0.77; 1.12]
Li,2005	0.15	[−0.05; 0.36]
Li,2009	0.34	[0.13; 0.55]
Li,2012	0.23	[−0.11; 0.57]
Liu,2008	0.30	[−0.17; 0.78]
Lu,2014	0.03	[−0.10; 0.16]
Lu,2020 data 1	0.04	[−0.24; 0.31]
Lu,2020 data 2	0.15	[−0.05; 0.35]
Mao,2013 data 1	−0.17	[−0.45; 0.11]
Mao,2013 data 2	−0.36	[−0.82; 0.10]
Mao,2013 data 3	0.04	[−0.26; 0.35]
Mao,2013 data 4	−0.24	[−0.68; 0.20]
Min,2014	0.69	[0.45; 0.92]
Shi,2009 data 1	0.11	[−0.25; 0.46]
Shi,2009 data 2	−0.03	[−0.36; 0.30]
Sun,2005	0.13	[−0.16; 0.41]
Takagi,2002	−0.52	[−0.73; −0.31]
Wang,2004 data 1	0.38	[0.10; 0.65]
Wang,2004 data 2	0.06	[−0.20; 0.32]
Wang,2006	0.15	[−0.05; 0.36]
Wang,2009	−0.01	[−0.25; 0.22]
Wang,2010	0.10	[−0.10; 0.29]
Wang,2012	0.99	[0.64; 1.35]
Wang,2013	0.21	[−0.19; 0.61]
Wang,2016	−0.04	[−0.28; 0.20]
Wang,2018	−0.08	[−0.38; 0.22]
Wang,2021 data 1	−0.21	[−0.57; 0.15]
Wang,2021 data 2	−0.10	[−0.42; 0.22]
Wang1,2006	0.19	[−0.06; 0.45]
Wu,2007	0.24	[−0.17; 0.65]
Wu1,2007	0.35	[0.03; 0.66]
Xia,2011 data 1	0.20	[−0.06; 0.46]
Xia,2011 data 2	0.11	[−0.23; 0.44]
Xiao,2004	0.04	[−0.11; 0.19]
Xiao,2005	0.03	[−0.09; 0.16]
Xiao,2012	0.10	[−0.18; 0.38]
Xue,2012	0.28	[0.07; 0.49]
Ya,2017	0.85	[0.44; 1.27]
Ya,2017 data 2	−1.21	[−1.64; −0.79]
Yamakawa,2004	−0.13	[−0.40; 0.13]
Yang,2011	0.08	[−0.19; 0.35]
Yao,2016a data 1	−0.19	[−0.46; 0.09]
Yao,2016a data 2	0.30	[0.00; 0.60]
Yao,2016b data 1	−0.16	[−0.42; 0.09]
Yao,2016b data 2	0.14	[−0.10; 0.38]
Yao,2018	−0.13	[−0.33; 0.07]

(*Continued*)

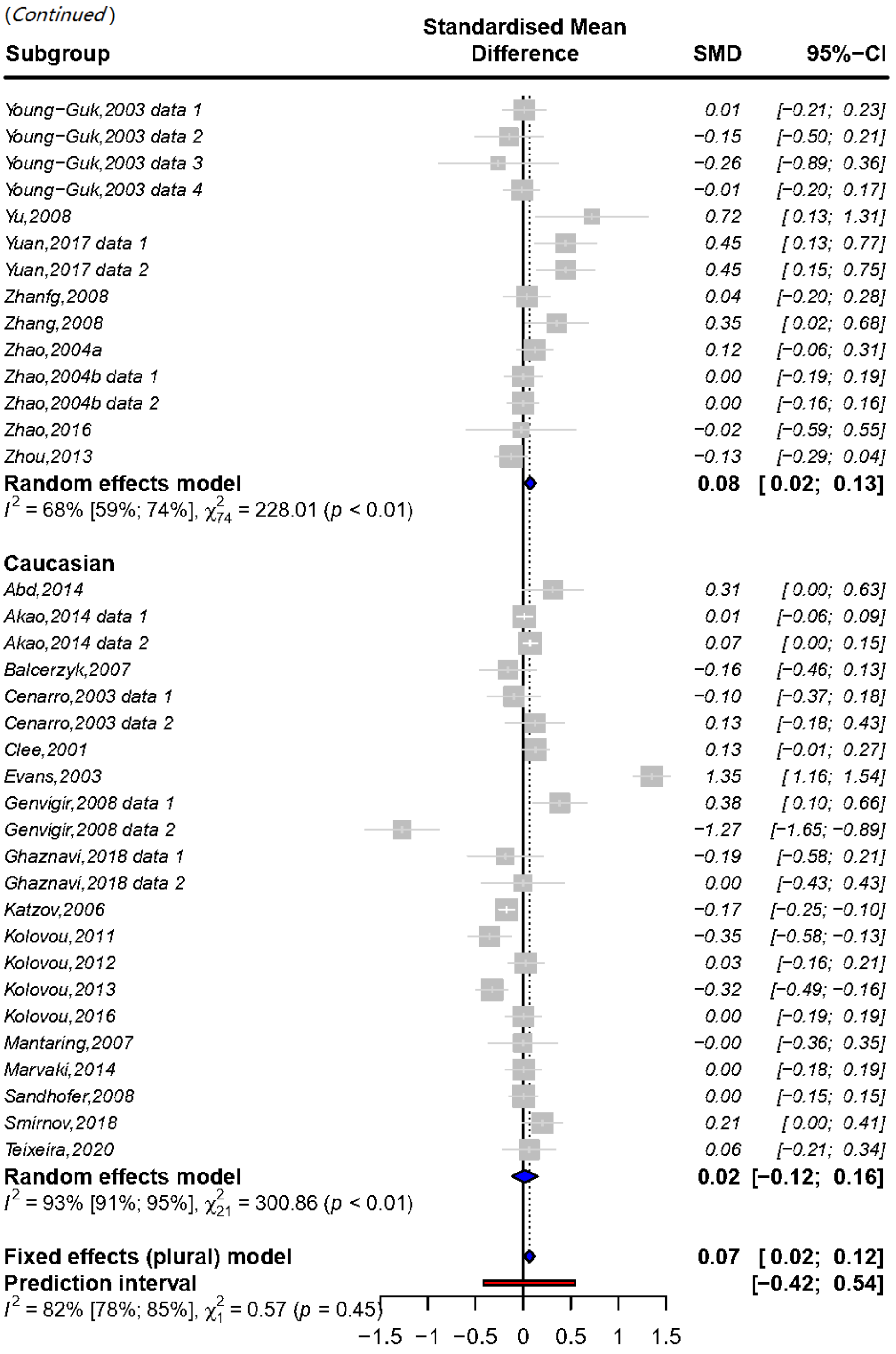

Supplementary Figure S7. The effects of R219K on TG level (**Asian:** SMD = 0.08, 95%CI: 0.02 ~ 0.13; z = 2.65, P < 0.01; **Caucasian:** SMD = 0.02, 95%CI: -0.12 ~ 0.16; z = 0.27, P = 0.78; and **overall:** SMD = 0.07, 95%CI: 0.02 ~ 0.12; z = 2.56, P = 0.01.)

2.4.4 Meta-regression analysis (under recessive model)

Moderator	Studies	Coefficient (β)	SE	95% CI	z	P
Intercept		-65.05	36.50	-136.59 ~ 6.49	-1.78	0.07
Publication time	103	0.03	0.02	-0.00 ~ 0.07	1.75	0.08
Race: Caucasian [a]	25	0.45	0.26	-0.27 ~ 0.77	0.94	0.35
Sample size	103	< 0.01	< 0.01	-0.01 ~ 0.01	-0.03	0.97
Health condition [b]				Q = 1.35, df = 2, P = 0.51		
Patients	46	-0.23	0.26	-0.74 ~ 0.28	-0.89	0.37
Random	36	0.01	0.27	-0.53 ~ 0.55	0.04	0.97
Sex	97	-1.57	0.32	-2.20 ~ -0.93	-4.85	**< 0.01**
Age	100	< 0.01	0.01	-0.02 ~ 0.02	0.24	0.81
BMI	73	-0.02	0.05	-0.12 ~ 0.07	-0.53	0.60
NOS	103	0.27	0.13	0.03 ~ 0.52	2.17	**0.03**

Supplementary Table S5. Meta-Regression analysis for relationship between R219K and TG under recessive model. Test of the model: $Q_M = 32.88$, df = 9, P < 0.01; Goodness of fit test: $Q_G = 211.94$, df = 59, P < 0.01. [a] Samples of Asian as reference; [b] Samples including patients and controls (mix) set as reference; BMI, body mass index; NOS, Newcastle-Ottawa Scale (NOS) scale; Bold indicates statistically significant (P < 0.05).

References

1 Çoban, N. et al. Gender specific association of ABCA1 gene R219K variant in coronary disease risk through interactions with serum triglyceride elevation in Turkish adults. Anatolian Journal of Cardiology/Anadolu Kardiyoloji Dergisi **14** (2014).

2 Delgado-Lista, J. et al. ABCA1 gene variants regulate postprandial lipid metabolism in healthy men. Arteriosclerosis, thrombosis, and vascular biology **30**, 1051-1057, doi:10.1161/atvbaha.109.202580 (2010).

3 Evans, D. & Beil, F. U. The association of the R219K polymorphism in the ATP-binding cassette transporter 1 (ABCA1) gene with coronary heart disease and hyperlipidaemia. Journal of Molecular Medicine-Jmm **81**, 264-270, doi:10.1007/s00109-003-0426-y (2003).

4 Fawzy, M. S. et al. Functional and Structural Impact of ATP-Binding Cassette Transporter A1 R219K and I883M Gene Polymorphisms in Obese Children and Adolescents. Molecular diagnosis & therapy **19**, 221-234, doi:10.1007/s40291-015-0150-7 (2015).

5 Ghaznavi, H., Aali, E. & Soltanpour, M. S. Association Study of the ATP - Binding Cassette Transporter A1 (ABCA1) Rs2230806 Genetic Variation with Lipid Profile and Coronary Artery Disease Risk in an Iranian Population. Open access Macedonian journal of medical sciences **6**, 274-279, doi:10.3889/oamjms.2018.063 (2018).

6 Kitjaroentham, A., Hananantachai, H., Tungtrongchitr, A., Pooudong, S. & Tungtrongchitr, R. R219K polymorphism of ATP binding cassette transporter A1 related with low HDL in overweight/obese Thai males. Archives of medical research **38**, 834-838, doi:10.1016/j.arcmed.2007.06.010 (2007).

7 Kolovou, G. et al. Cholesteryl ester transfer protein and ATP-binding cassette transporter A1 genotype alter the atorvastatin and simvastatin efficacy: time for genotype-guided therapy? Angiology **64**, 266-272 (2013).

8 Kolovou, V. et al. Effect of ATP-binding cassette transporter A1 (ABCA1) gene polymorphisms on plasma lipid variables and common demographic parameters in Greek nurses. The open cardiovascular medicine journal **10**, 233 (2016).

9 Kolovou, V. et al. Association of gender, ABCA1 gene polymorphisms and lipid profile in Greek young nurses. Lipids in health and disease **11**, doi:10.1186/1476-511x-11-62 (2012).

10 Manresa, J. M. et al. Relationship of classical and non-classical risk factors with genetic variants relevant to coronary heart disease. Eur J Cardiovasc Prev Rehabil **13**, 738-744, doi:10.1097/01.hjr.0000224484.80349.3f (2006).

11 Mantaring, M., Rhyne, J., Ho Hong, S. & Miller, M. Genotypic variation in ATP-binding cassette transporter-1 (ABCA1) as contributors to the high and low high-density lipoprotein-cholesterol (HDL-C) phenotype. Translational research : the journal of laboratory and clinical medicine **149**, 205-210, doi:10.1016/j.trsl.2006.11.007 (2007).

12 Marvaki, A. et al. Impact of 3 Common ABCA1 Gene Polymorphisms on Optimal vs Non-Optimal Lipid Profile in Greek Young Nurses. The open cardiovascular medicine journal **8**, 83-87, doi:10.2174/1874192401408010083 (2014).

13 Mokuno, J. et al. ATP-binding cassette transporter A1 (ABCA1) R219K (G1051A, rs2230806) polymorphism and serum high-density lipoprotein cholesterol levels in a large Japanese population: cross-sectional data from the Daiko Study. Endocrine journal **62**, 543-549, doi:10.1507/endocrj.EJ14-0577 (2015).

14 Sandhofer, A. et al. The influence of two variants in the adenosine triphosphate-binding cassette transporter 1 gene on plasma lipids and carotid atherosclerosis. Metabolism **57**, 1398-1404, doi:10.1016/j.metabol.2008.05.009 (2008).

15 Srinivasan, S. R., Li, S., Chen, W., Boerwinkle, E. & Berenson, G. S. R219K polymorphism of the ABCA1 gene and its modulation of the variations in serum high-density lipoprotein cholesterol and triglycerides related to age and adiposity in white versus black young adults. The Bogalusa heart study. Metabolism: clinical and experimental **52**, 930-934, doi:10.1016/s0026-0495(03)00076-3 (2003).

16 Teixeira, M. D., Tureck, L. V., Nascimento, G. A. D., Souza, R. L. R. & Furtado-Alle, L. Is it possible ABC transporters genetic variants influence the outcomes of a weight-loss diet in obese women? Genetics and Molecular Biology **43**, e20190326, doi:10.1590/1678-4685-GMB-2019-0326 (2020).

17 Yamakawa-Kobayashi, K. et al. Associations between serum high-density lipoprotein cholesterol or apolipoprotein AI levels and common genetic variants of the ABCA1 gene in Japanese school-aged children. Metabolism: clinical and experimental **53**, 182-186, doi:10.1016/j.metabol.2003.08.009 (2004).

18 Ko, Y. G. et al. Association of R219K Polymorphism in the ABCA1 Gene with Plasma Lipid Levels and Coronary Artery Disease in Koreans. Korean Circulation Journal **33** (2003).

19 Rugge, B. et al. Screening and treatment of subclinical hypothyroidism or hyperthyroidism. (2011).

20 Yao, M.-H. et al. Association between Polymorphisms and Haplotype in the ABCA1 Gene and Overweight/Obesity Patients in the Uyghur Population of China. International journal of environmental

research and public health **13**, doi:10.3390/ijerph13020220 (2016).
21 Hozo, S. P., Djulbegovic, B. & Hozo, I. Estimating the mean and variance from the median, range, and the size of a sample. BMC medical research methodology **5**, 13 (2005).
22 Graffelman, J. Exploring Diallelic Genetic Markers: The HardyWeinberg Package. 2015 **64**, 23, doi:10.18637/jss.v064.i03 (2015).
23 Abd El-Aziz, T. A., Mohamed, R. H. & Hagrass, H. A. Increased risk of premature coronary artery disease in Egyptians with ABCA1 (R219K), CETP (TaqIB), and LCAT (4886C/T) genes polymorphism. J Clin Lipidol **8**, 381-389, doi:10.1016/j.jacl.2014.06.001 (2014).
24 Sun, M. et al. Distributional characteristics of adenosine triphosphate binding cassette transport protein A1 gene R219K polymorphism and association with blood lipid level in healthy Han and Uygur population in Xinjiang. Journal of Chinese practical diagnosis and therapy **25** (2011).
25 Ya, L. & Lu, Z. Differences in ABCA1 R219K polymorphisms and serum indexes in Alzheimer and Parkinson Diseases in Northern China. Medical science monitor: international medical journal of experimental and clinical research **23**, 4591 (2017).
26 Katzov, H. et al. Quantitative trait loci in ABCA1 modify cerebrospinal fluid amyloid-β 1-42 and plasma apolipoprotein levels. Journal of human genetics **51**, 171-179 (2006).
27 Delgado-Lista, J. et al. ABCA1 gene variants regulate postprandial lipid metabolism in healthy men. Arteriosclerosis, thrombosis, and vascular biology **30**, 1051-1057 (2010).
